# Supplementary material for: Potent and selective covalent inhibition of the papain-like protease from SARS-CoV-2
Source: Nat Commun. 2023 Mar 28;14:1733. doi: 10.1038/s41467-023-37254-w (PMC10044120; doi:10.1038/s41467-023-37254-w)
Supplement: Supplementary file 1 — Supplementary Information [file 41467_2023_37254_MOESM1_ESM.pdf]

Supplementary Information for

Potent and Selective Covalent Inhibition of the Papain-like Protease from SARS-CoV-2

Brian C. Sanders,[1,†,*] Suman Pokhrel,[2,3,†] Audrey D. Labbe,[1] Irimpan I. Mathews,[4] Connor J. Cooper,[1] Russell B. Davidson,[1] Gwyndalyn Phillips,[5] Kevin L. Weiss,[5] Qiu Zhang,[5] Hugh O'Neill,[5] Manat Kaur,[6] Jurgen G. Schmidt,[7] Walter Reichard,[8] Surekha Surendranathan,[9] Jyothi Parvathareddy,[9] Lexi Phillips,[10] Christopher Rainville,[11] David E. Sterner,[11] Desigan Kumaran,[12] Babak Andi,[13] Gyorgy Babnigg,[14,15] Nigel W. Moriarty,[16] Paul D. Adams,[16,17] Andrzej Joachimiak,[14,18] Brett L. Hurst,[10] Suresh Kumar,[11] Tauseef R. Butt,[11] Colleen B. Jonsson,[8] Lori Ferrins,[19] Soichi Wakatsuki,[2,3,*] Stephanie Galanie,[1] Martha S. Head,[20,21] and Jerry M. Parks[1,*]

[1] Biosciences Division, Oak Ridge National Laboratory, Oak Ridge, Tennessee, USA
[2] Department of Chemical and Systems Biology, Stanford University School of Medicine, Stanford, California, USA
[3] Biological Sciences Division, SLAC National Accelerator Laboratory, Menlo Park, California, USA
[4] Stanford Synchrotron Radiation Lightsource, Menlo Park, California, USA
[5] Neutron Scattering Division, Oak Ridge National Laboratory, Oak Ridge, Tennessee, USA
[6] Department of Structural Biology, Stanford University School of Medicine, Stanford, California, USA
[7] B-11 Bioenergy and Biome Sciences, Bioscience Division, Los Alamos National Laboratory, Los Alamos, New Mexico, USA
[8] Department of Microbiology, Immunology and Biochemistry, University of Tennessee Health Science Center, Memphis, Tennessee, USA
[9] Regional Biocontainment Laboratory, University of Tennessee Health Science Center, Memphis, TN, USA
[10] Institute for Antiviral Research, Department of Animal, Dairy, and Veterinary Sciences, Utah State University; Logan, UT, USA
[11] Progenra Inc., Malvern, Pennsylvania, USA
[12] Biology Department, Brookhaven National Laboratory, Upton, New York, USA
[13] Center for BioMolecular Structure, National Synchrotron Light Source II, Brookhaven National Laboratory, Upton, New York, USA
[14] Center for Structural Genomics of Infectious Diseases, Consortium for Advanced Science and Engineering, University of Chicago, Chicago, Illinois, USA
[15] Biosciences Division, Argonne National Laboratory, Argonne, Illinois, USA
[16] Molecular Biosciences and Integrated Bioimaging, Lawrence Berkeley National Laboratory, Berkeley, California, USA
[17] Department of Bioengineering, University of California, Berkeley, CA, USA
[18] Structural Biology Center, X-ray Science Division, Argonne National Laboratory, Argonne, IL, USA. Department of Chemistry, University of Chicago, Chicago, Illinois, USA
[19] Department of Chemistry and Chemical Biology, Northeastern University, Boston, Massachusetts, USA
[20] Joint Institute for Biological Sciences, Oak Ridge National Laboratory, Oak Ridge, Tennessee USA
[21] Computing and Computational Sciences Directorate, Oak Ridge National Laboratory, Oak Ridge, Tennessee USA

[†] These authors contributed equally to this work.

[*]Correspondence and requests for materials should be addressed to sandersbc@ornl.gov, soichi.wakatsuki@stanford.edu, or parksjm@ornl.gov.

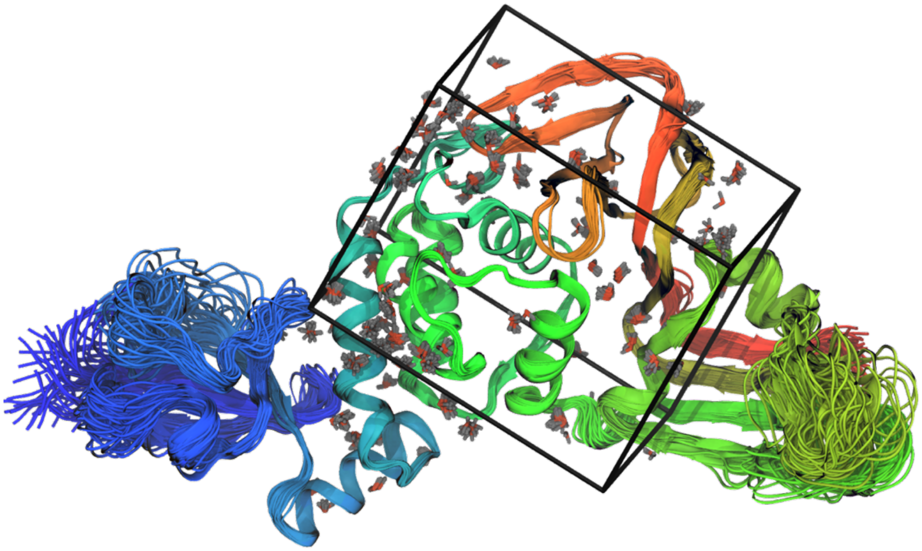

Supplementary Figure 1. Superposition of 50 ensemble models generated from crystallographic data for PDB entry 7JIR.[14] The protein is shown in spectrum coloring (blue = N-terminus, red = C-terminus). The docking box is shown in thick black lines. Conserved water molecules are shown in stick representation. Ser111 was mutated back to Cys in all models prior to docking.

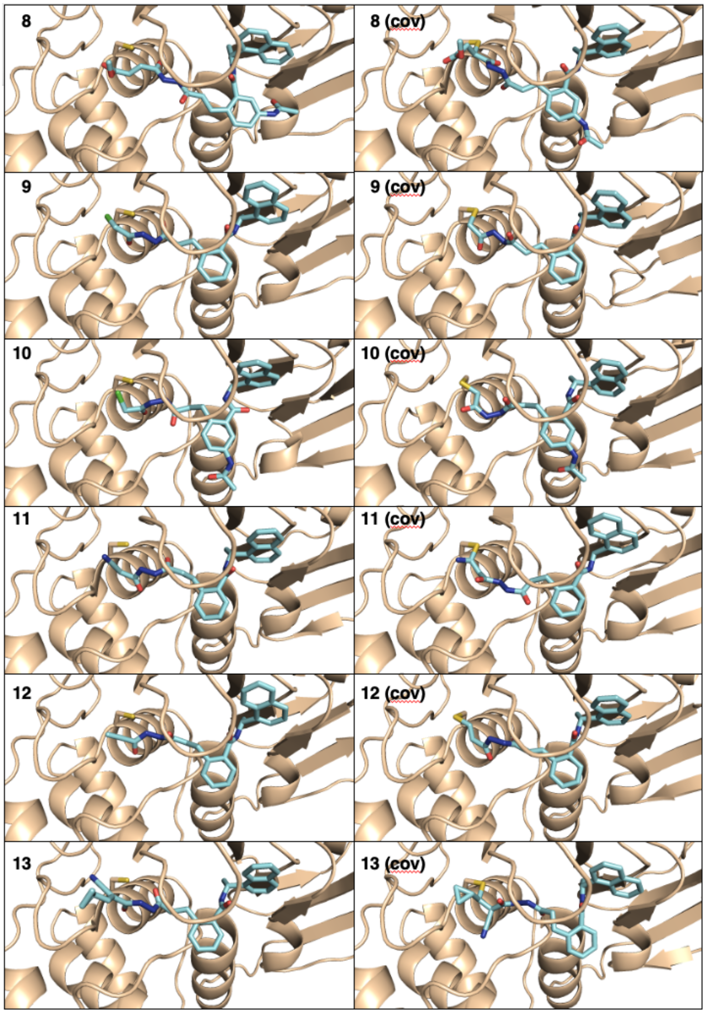

Supplementary Figure 2. Docked poses of compounds **8-13**. *Left*: noncovalent, *right*: covalent.

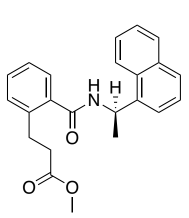
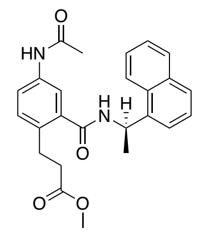
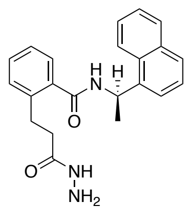
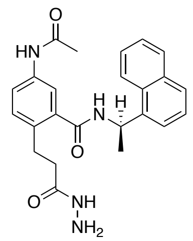

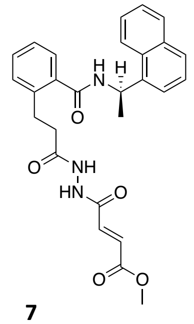
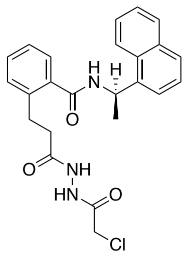
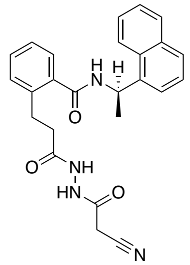
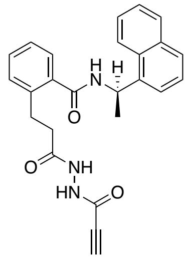
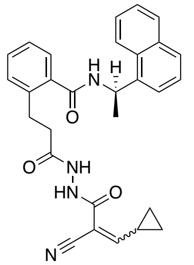

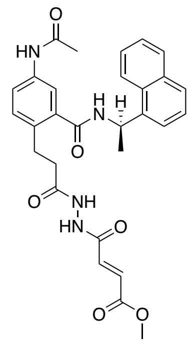
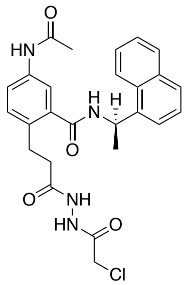
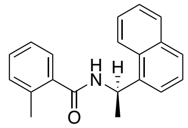
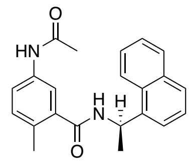

**Compound number**	**Noncovalent score (RMSD)**	**Covalent score (RMSD)**
**3**	-11.8 (0.6)	n/a
**4**	-12.5 (0.9)	n/a
**5**	-12.0 (0.8)	n/a
**6**	-12.9 (0.8)	n/a
**7**	-12.8 (1.1)	-12.5 (0.8)
**9**	-12.7 (1.0)	-11.8 (0.7)
**11**	-13.3 (0.7)	-10.1 (1.5)
**12**	-12.3 (1.1)	-12.1 (0.7)
**13**	-13.2 (1.2)	n/a (>2.0)
**8**	n/a (n/a)	-13.3 (1.0)
**10**	-11.4 (1.3)	-12.3 (1.0)
**14**	-10.1 (0.5)	n/a
**15**	-10.9 (0.9)	n/a

Supplementary Figure 3. Summary of docking results for experimentally tested compounds. Docking scores are in kcal/mol and MCS-RMSD values are in Å. Additional docking data for all candidate inhibitors are provided in **Supplementary Data File 1**.

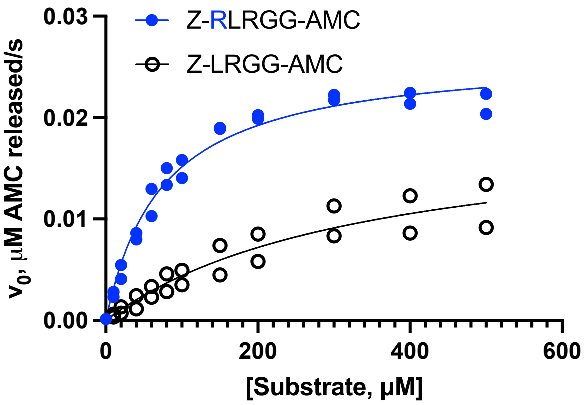

	Z-RLRGG-AMC	Z-LRGG-AMC
**Michaelis-Menten**		
**Best-fit values**		
V_{max}	0.026	0.020
K_m	74	345
**Goodness of Fit**		
Degrees of Freedom	22	22
R^2	0.98	0.91

Supplementary Figure 4. Michaelis-Menten kinetics data for Z-RLRGG-AMC and Z-LRGG-AMC substrates. Data points represent mean values for n = 2 independent experiments. Units in the table correspond to those in the x and y axes of the plot, i.e., µM/s for V_{max} and µM for K_m. Source data are provided as a Source Data file.

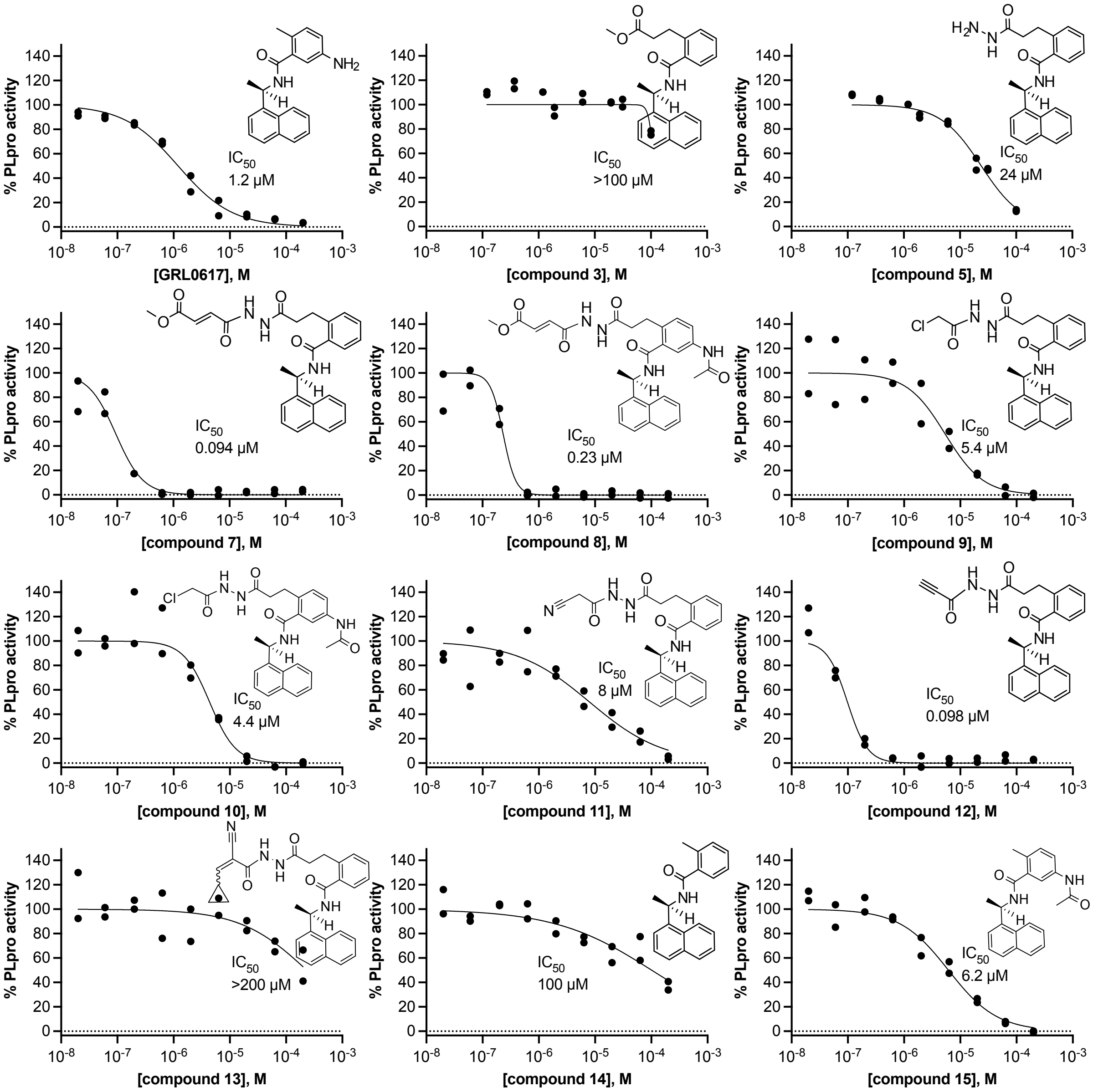

Supplementary Figure 5. Fluorogenic peptide activity assay after 30 min preincubation with inhibitor candidates. Data points represent the mean of $n = 2$ independent samples. IC_{50} is the concentration at which 50% inhibition was observed. Curve is the nonlinear regression to the normalized inhibitor dose response equation. Source data are provided as a Source Data file.

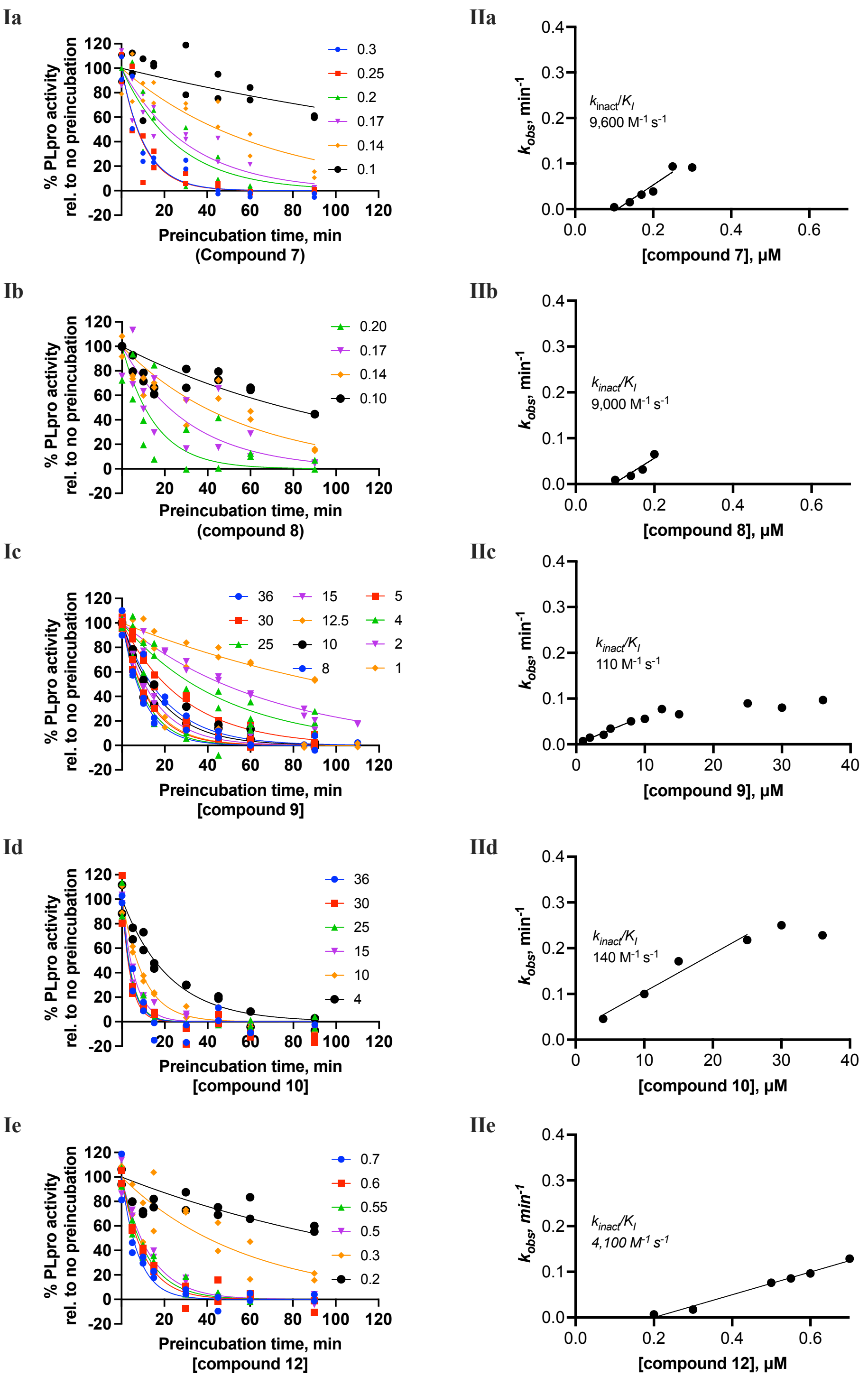

Supplementary Figure 6. Time-dependent characterization with fluorogenic peptide assay. Data in the left column (**Ia-e**) are initial rates determined at various inhibitor concentrations (μM) with compound number in the *x*-axis label and preincubation times normalized to no preincubation. Data are shown for n = 2 independent samples. Curves are nonlinear regressions of the exponential decay equation to the data. Data in the right column (**IIa-e**) are the mean k_{obs} values determined from **Ia-e**. Each line represents the linear regression yielding as its slope the second-order rate constant (k_{inact}/K_I). Source data are provided as a Source Data file.

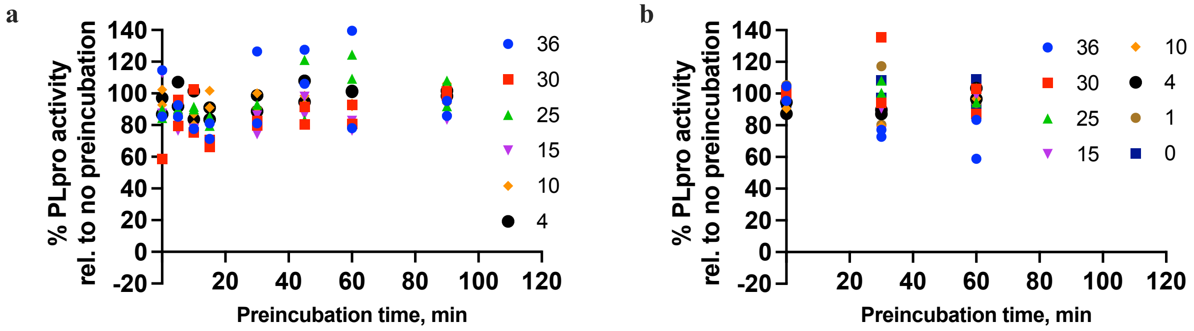

Supplementary Figure 7. Time-dependent characterization with fluorogenic peptide assay. (**a**) compound **11**, (**b**) compound **13**. Data are from $n = 2$ independent samples. Source data are provided as a Source Data file.

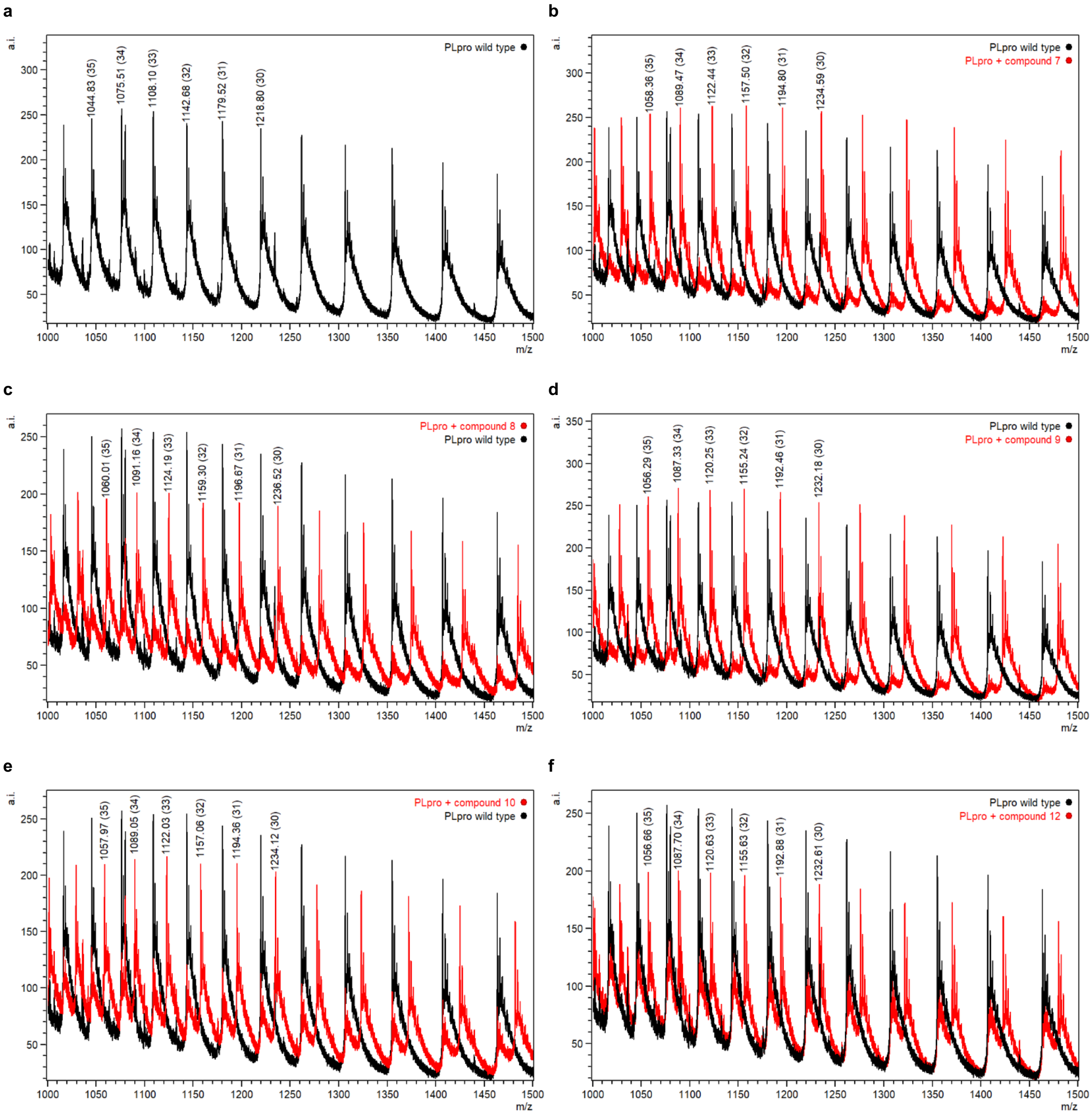

Supplementary Figure 8. Intact protein ESI-MS spectra of (**a**) PLpro (black), and (**b-f)**, PLpro incubated with compounds indicated in the figure legends (red). a.i., arbitrary intensity; m/z, mass-to-charge ratio. Spectral peaks used for protein monoisotopic mass deconvolution are annotated with their monoisotopic masses and, in parentheses, charge states.

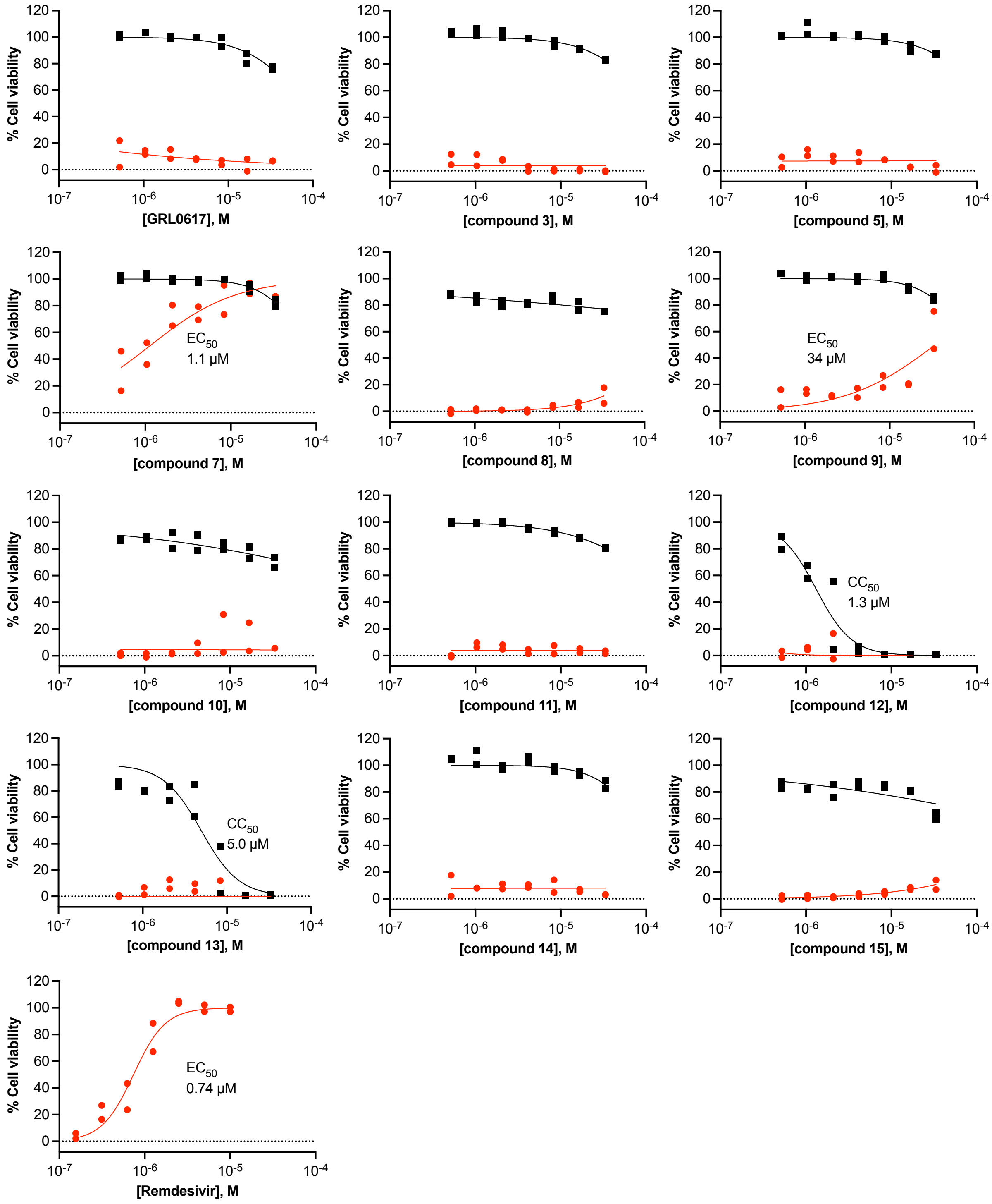

Supplementary Figure 9. Cell viability in Vero E6 cells for uninfected cells pretreated with indicated compound (black) and SARS-CoV-2-infected cells pretreated with indicated compound (red). Data are the mean of n = 2 independent samples. EC_{50} is the concentration at which 50% effect was observed, CC_{50} is the concentration at which 50% cytotoxicity was observed. Curves are the nonlinear regression to the normalized dose response equation. Source data are provided as a Source Data file.

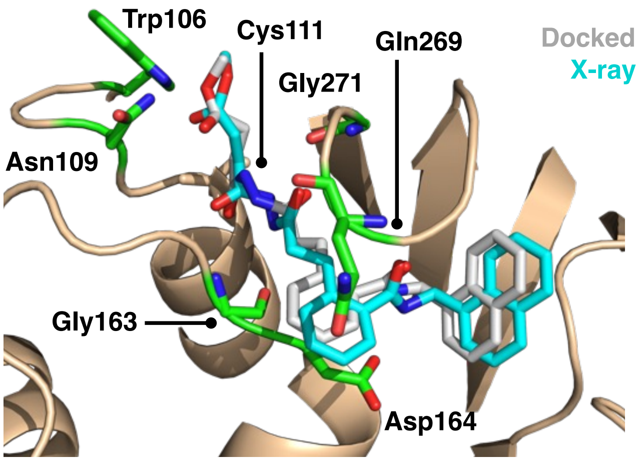

Supplementary Figure 10. Superposition of the covalently docked model of **7** (grey sticks) and the co-crystal structure of PLpro and **7** (cyan sticks). Selected residues in the binding site are labeled and shown as green sticks.

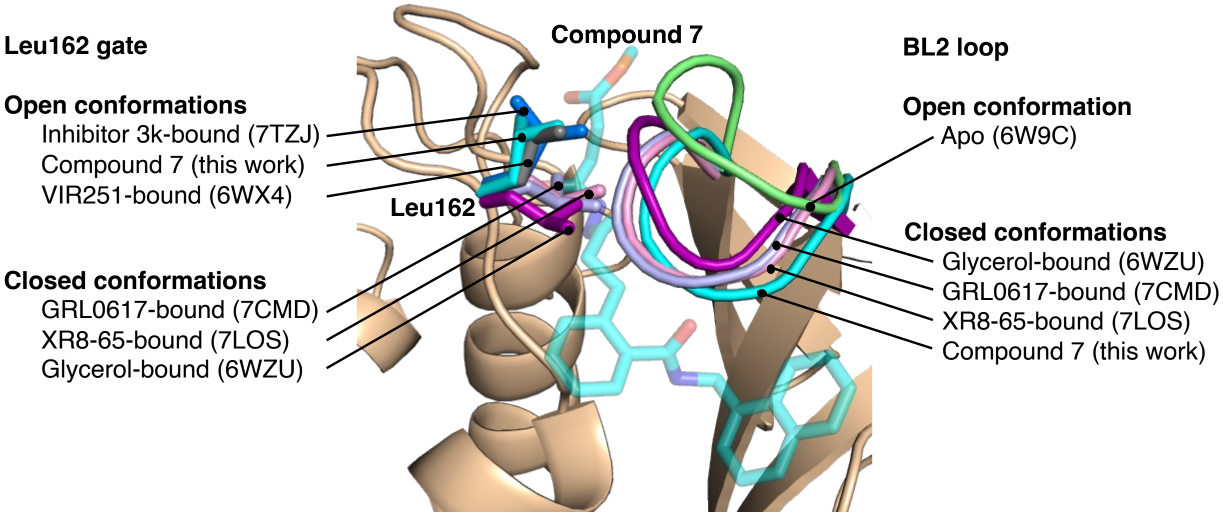

Supplementary Figure 11. Superposition of PLpro crystal structures highlighting different conformations of Leu162 (sticks) and the BL2 loop (cartoon): Ligand-free (PDB entry 6W9C, light green), glycerol-bound (PDB entry 6WZU, purple), inhibitor 3k-bound (PDB entry 7TZJ, marine blue), VIR251-bound (PDB entry 6WX4, grey), GRL0617-bound (PDB entry 7CMD, light purple), XR8-65-bound (PDB entry 7LOS, light pink) and compound **7**-bound (this work, cyan). Compound **7** is shown as semi-transparent cyan sticks.

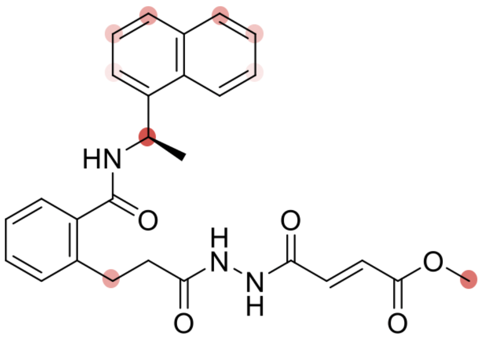

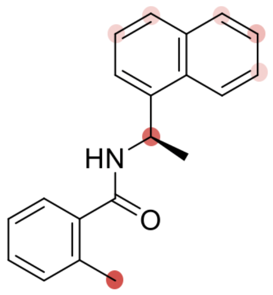

Supplementary Figure 12. MetaSite site-of-metabolism prediction for compound **7** (left) and **14** (right). The shade of the red circles indicates relative liability (darker = greater liability).

Supplementary Table 1. PLpro intact protein ESI-MS analysis.

**Compound**	**Deconvoluted monoisotopic PLpro + compound mass**	**Measured adduct mass**	**Predicted adduct mass**
- (wild-type PLpro)	36535 (FWHM 16)	-	-
**7**	37009 (FWHM 16)	474	473
**8**	37066 (FWHM 16)	531	530
**9**	36936 (FWHM 15)	401	401
**10**	36995 (FWHM 15)	460	458

FWHM = full-width half maximum

Supplementary Table 2. Deubiquitinase selectivity assay data.

DUB, enzyme conc. (substrate)	Compound								
	15			9			7		
	IC_{50} (μM)	Hill slope	SI[a]	IC_{50} (μM)	Hill slope	SI[a]	IC_{50} (μM)	Hill slope	SI[a]
PLpro, 50 nM (Ub-rhodamine110)	>30	0.376	N/A	1.96	0.705	N/A	0.076	1.06	N/A
PLpro, 10 nM (ISG15-CHOP2)	>30	0.969	N/A	20.2	0.817	N/A	0.037	1.34	N/A
USP30, 10 nM (Ub-rhodamine110)	>30	N/A	1	>30	N/A	15.3	>30	N/A	394.7
USP15, 1 nM (Ub-rhodamine110)	>30	N/A	1	>30	N/A	15.3	>30	N/A	394.7
USP8, 10 nM (Ub-rhodamine110)	>30	N/A	1	>30	N/A	15.3	>30	N/A	394.7
USP7, 5 nM (Ub-CHOP2)	>30	N/A	1	>30	N/A	15.3	>30	N/A	394.7
USP4, 10 nM (Ub-rhodamine110)	>30	N/A	1	>30	N/A	15.3	>30	N/A	394.7
USP2, 10 nM (Ub-rhodamine110)	>30	N/A	1	>30	N/A	15.3	>30	N/A	394.7
UCHL1, 10 nM (Ub-rhodamine110)	>30	N/A	1	>30	N/A	15.3	>30	N/A	394.7

[a] Selectivity index (SI) is represented as the fold change in selectivity for PLpro compared to DUB inhibition activity of other DUBs in the selectivity panel.

Supplementary Table 3. Summary of X-ray crystallography data collection and refinement

	PLpro compound **7**
**Crystallographic parameters**	
Space group	$I4_122$
Unit-cell dimensions	112.33 Å, 112.33 Å, 217.33 Å<br>90.0˚, 90.0˚, 90.0˚
**Data collection statistics**	
Resolution limits (Å)	39.1 – 3.10
No. of observed reflections	333876
No. of unique reflections	13010
Completeness<br>overall/outer shell	 99.8/99.8
CC1/2 (overall/outer shell)	99.8/75.2
R_{sym}^{a} (%)<br>overall/outer shell & os I/σ	 44.5/720.0 & 1.3
**Refinement statistics**	
Resolution limits (Å)	39.1-3.10
Number of reflections/%<br>(|F| > 2σ|F|)	12359/99.8
Reflections used for R_{free}	651
R_{factor}^{b} (%)	19.1
R_{free} (%)	25.3
Model contents/average B (Å^2)	
Protein atoms	2468/97.4
Ligand	35/81.7
Ions	8/101.9
Water molecules	42/71.6
RMS deviations	
Bond length (Å)	0.008
Bond angle (°)	1.79
Ramachandran<br>(analyzed/outliers)	311/5

Supplementary Table 4. Metabolic stability[a] in liver microsomes

	human		rat		mouse	
	half-life (min)	Cl_{int} (µL/min/mg)	half-life (min)	Cl_{int} (µL/min/mg)	half-life (min)	Cl_{int} (µL/min/mg)
**7**	50	138.8	46	151.2	13	539.7
**9**	7	974.9	13	514.7	5	1333.3
**14**	41	171.0	21	329.7	16	434.6
imipramine	>60	<115.5	5	1420.5	16	435.5
propranolol	>60	<115.5	3	2387.8	20	373.2
terfenadine	11	615.1	7	1053.6	8	851.4
verapamil	38	183.0	35	195.7	18	374.8

[a] mean of two replicates

Supplementary Table 5. Metabolic stability[a] in liver S9 fractions

	human		rat		mouse	
	half-life (min)	Cl_{int} (µL/min/mg)	half-life (min)	Cl_{int} (µL/min/mg)	half-life (min)	Cl_{int} (µL/min/mg)
**7**	60	38.9	31	75.5	9	266.4
**9**	3	689.4	16	140.4	4	544.0
**14**	>60	<38.5	17	137.5	18	128.4
imipramine	>60	<38.5	3	886.4	15	153.3
propranolol	>60	<38.5	3	765.7	6	356.6
terfenadine	16.0	144.5	8	290.8	14	160.1
verapamil	>60	<38.5	31	76.1	18	127.0

[a] mean of n = 2 replicates

Supplementary Table 6. Pharmacokinetic parameters for compound **7** in mouse plasma following a single IV administration in male ICR mice (dose: 3 mg/kg).

$t_{1/2}$ (h)	C_0 (ng/mL)	AUC_{last} (ng*hr/mL)	AUC_{inf} (ng*hr/mL)	V_{ss} (L/kg)	Cl (mL/min/kg)
0.06	63	4	5	35.21	11047.18

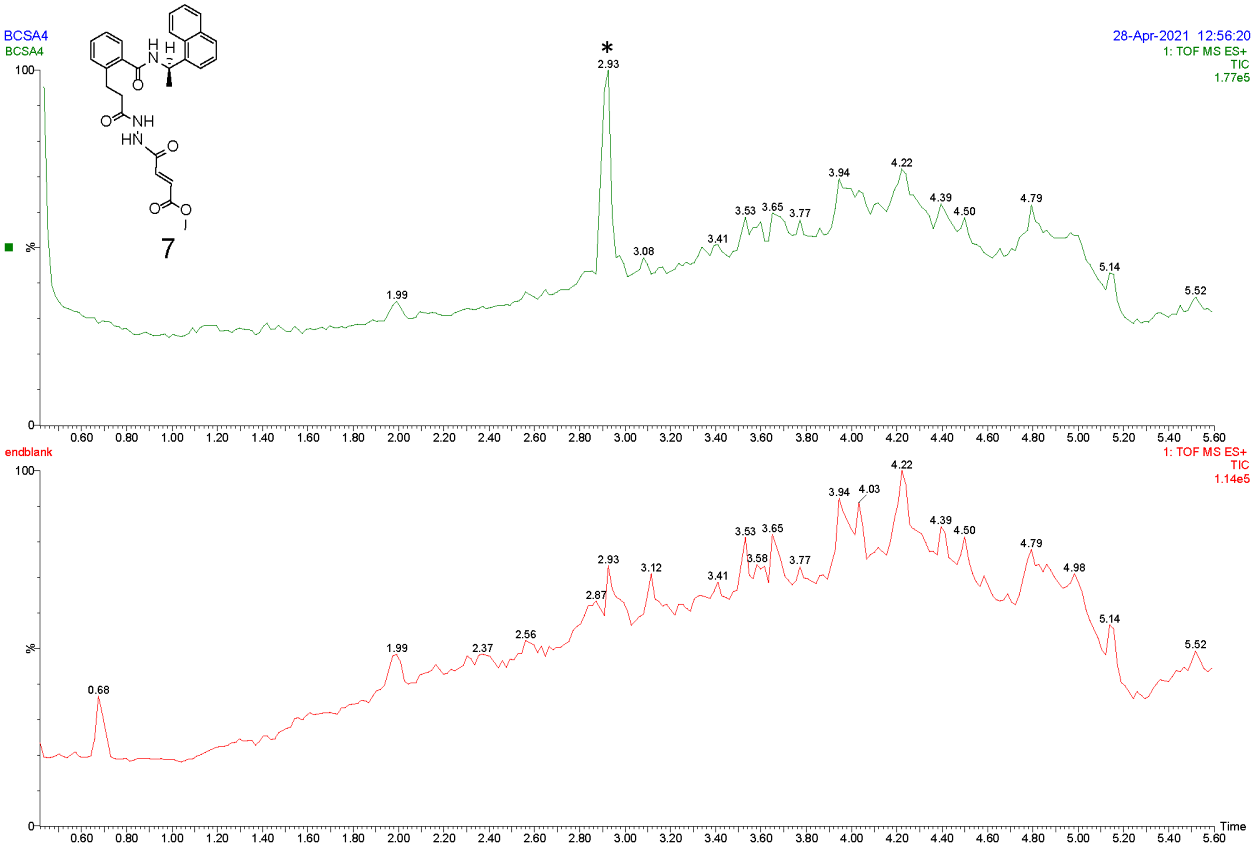

Supplementary Figure 13. LC/MS chromatogram of **7**. The top chromatogram (green) is the sample, and the bottom (red) is the background.

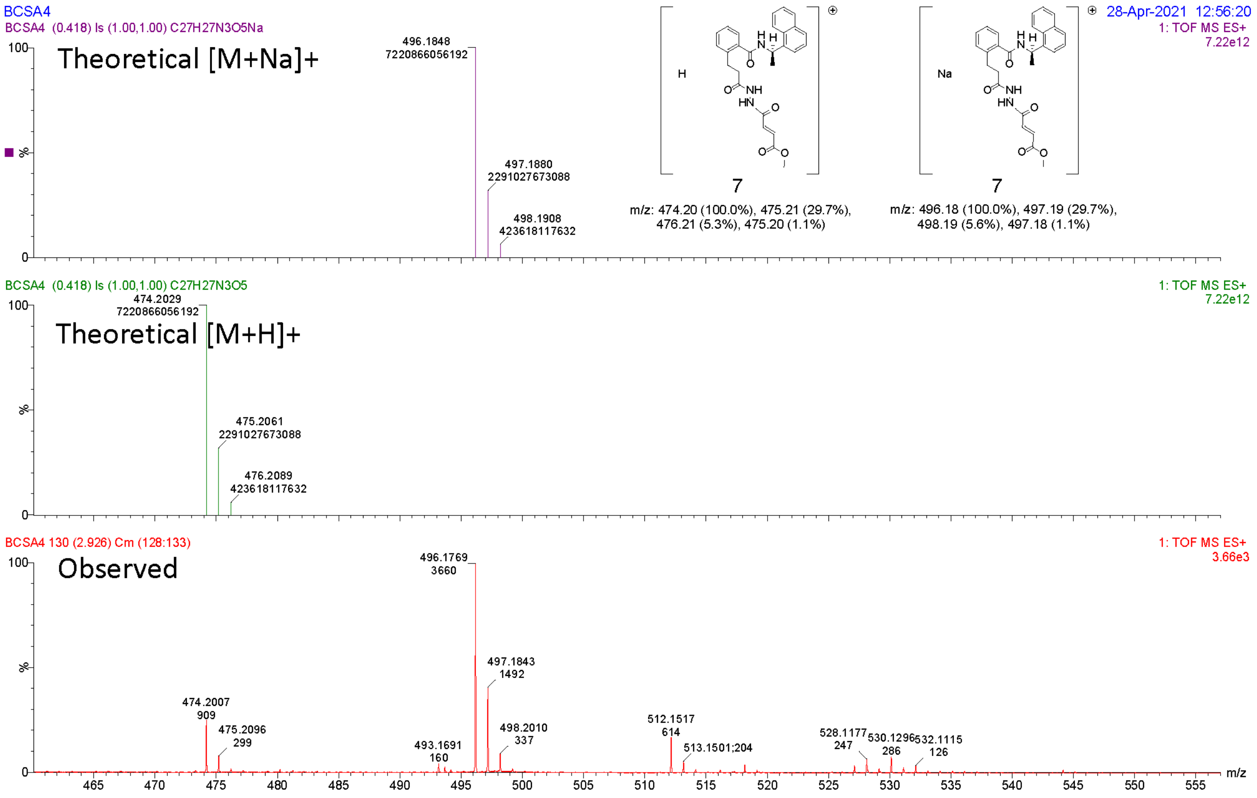

Supplementary Figure 14. HRMS of **7**.

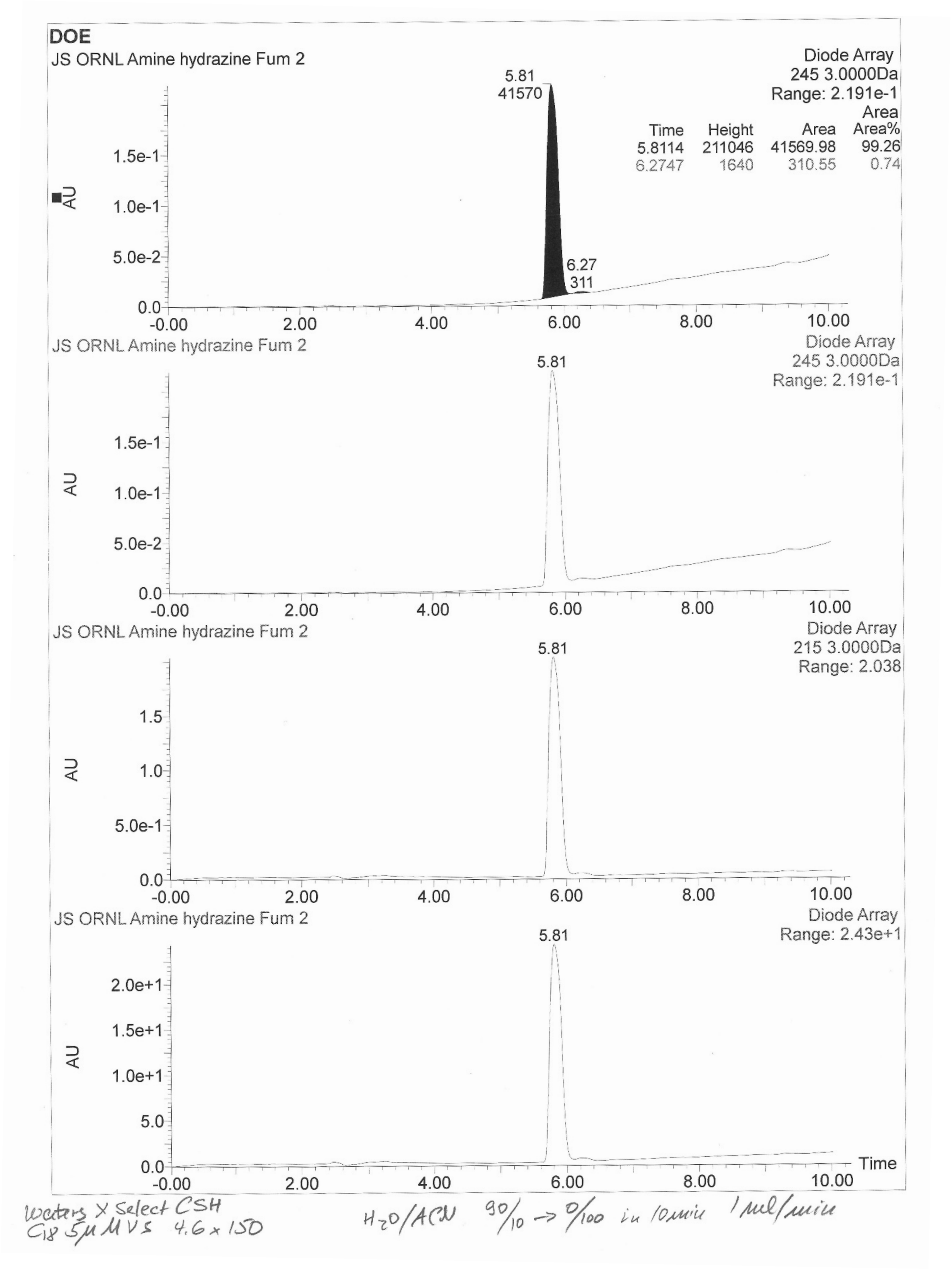

Supplementary Figure 15. HPLC chromatogram of **7**.

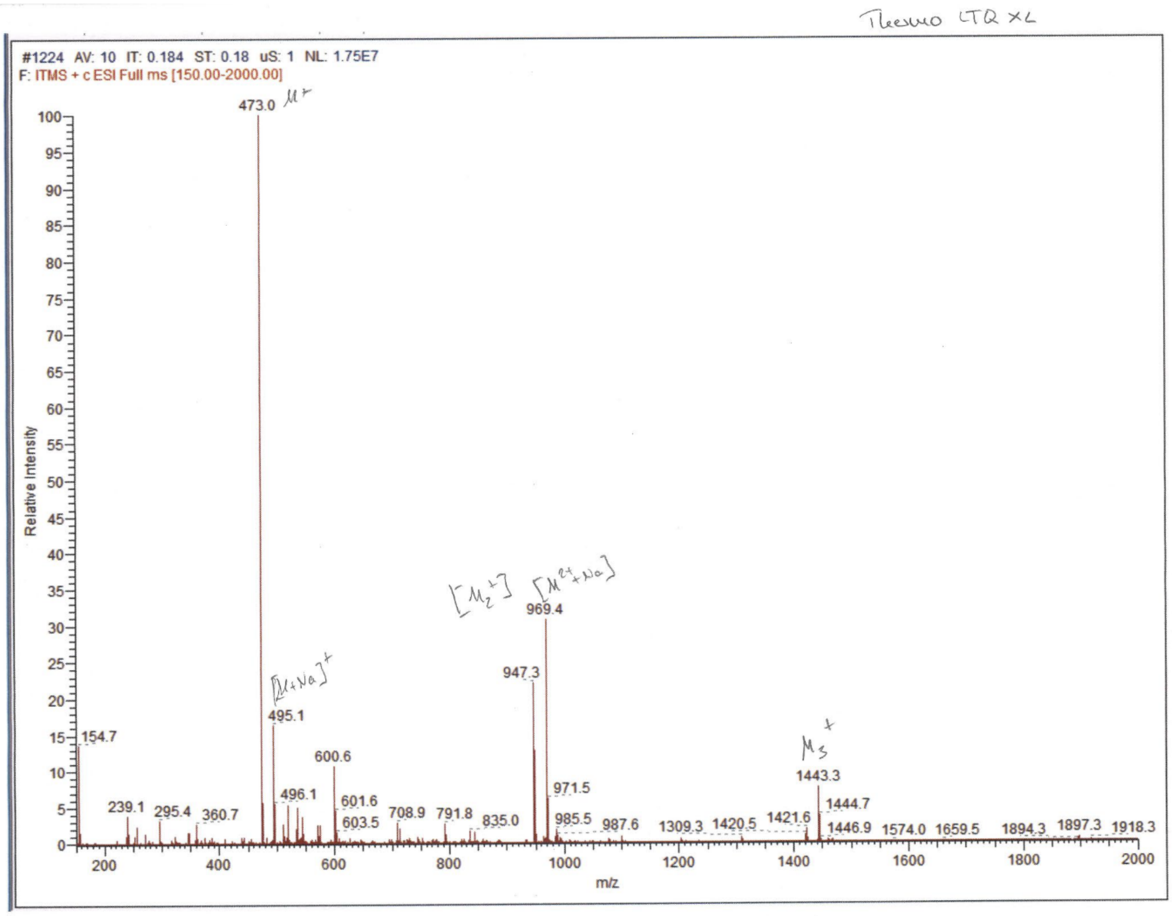

Supplementary Figure 16. LRMS of **7**.

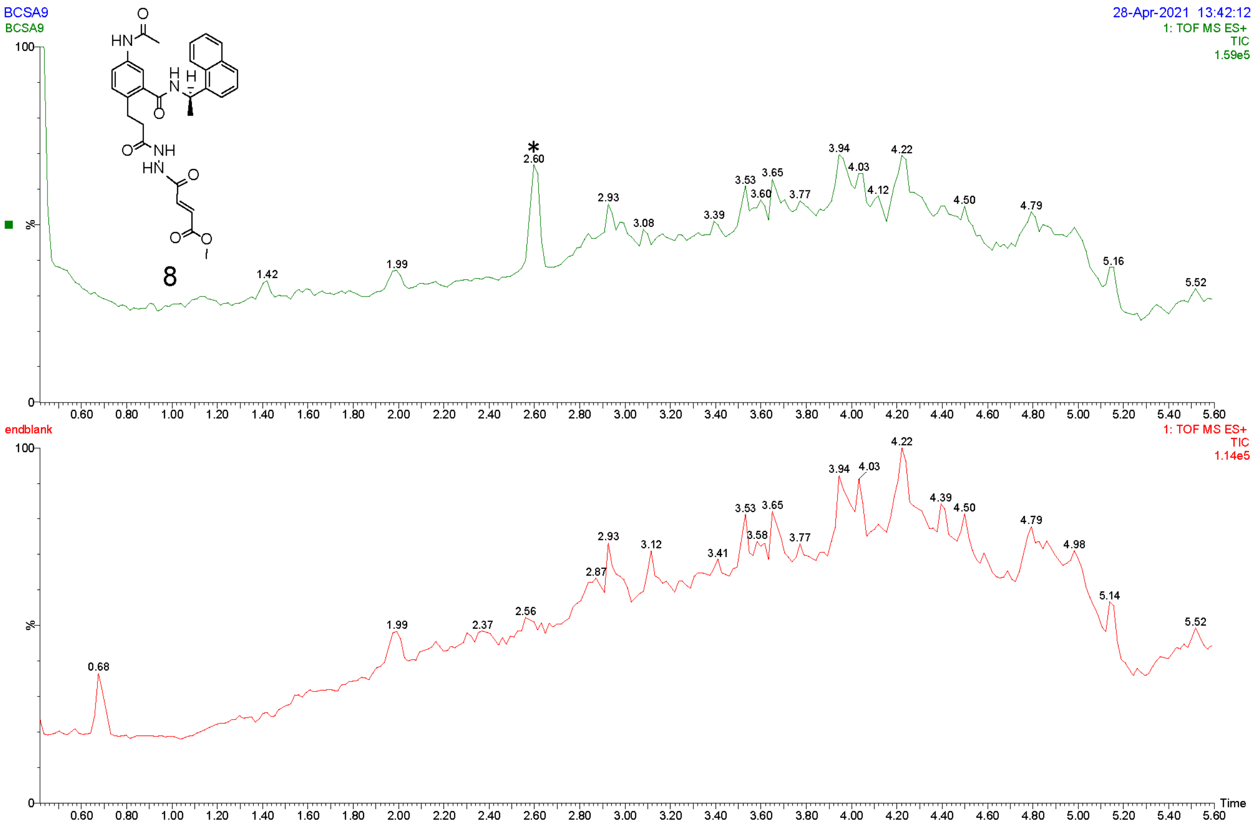

Supplementary Figure 17. LC/MS chromatogram of **8**. The top chromatogram (green) is the sample, and the bottom (red) is the background.

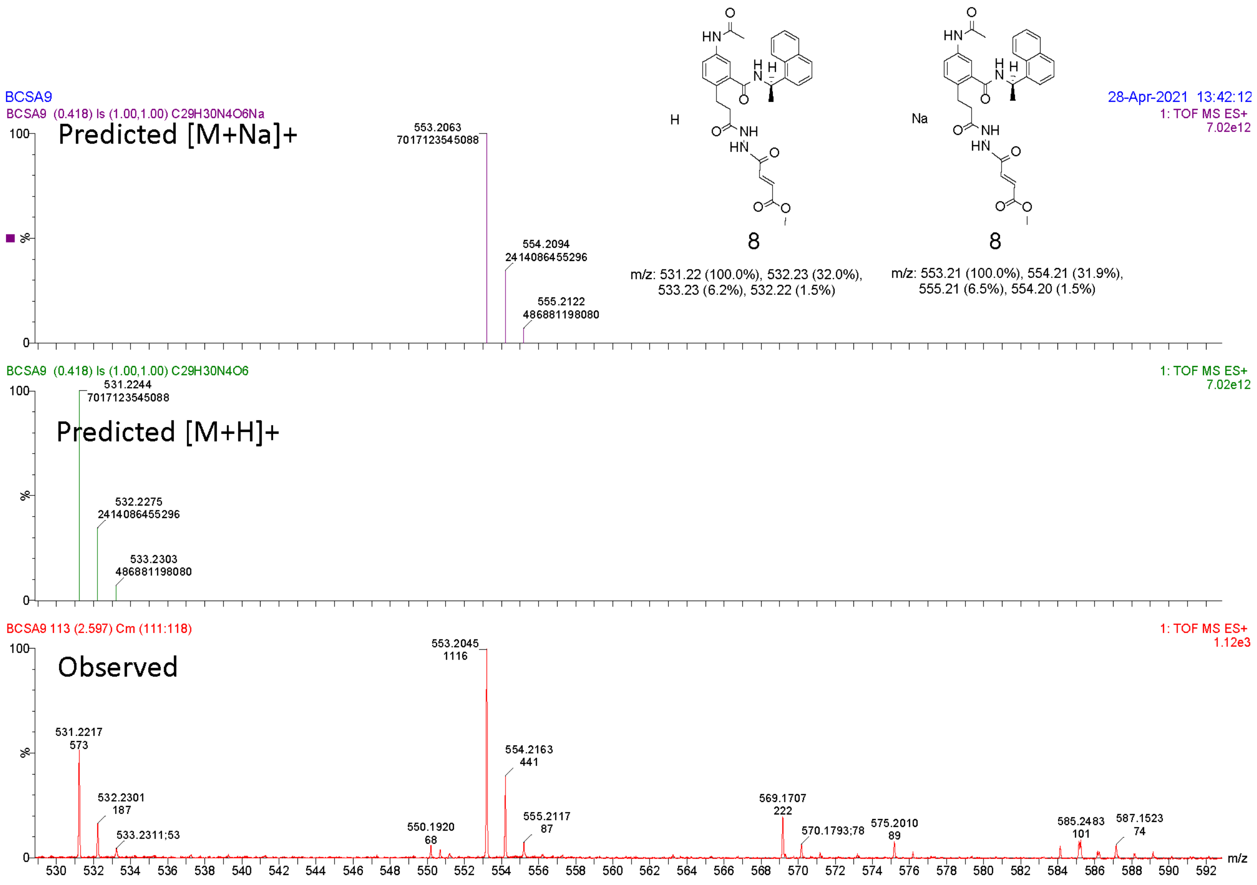

Supplementary Figure 18. HRMS of **8**.

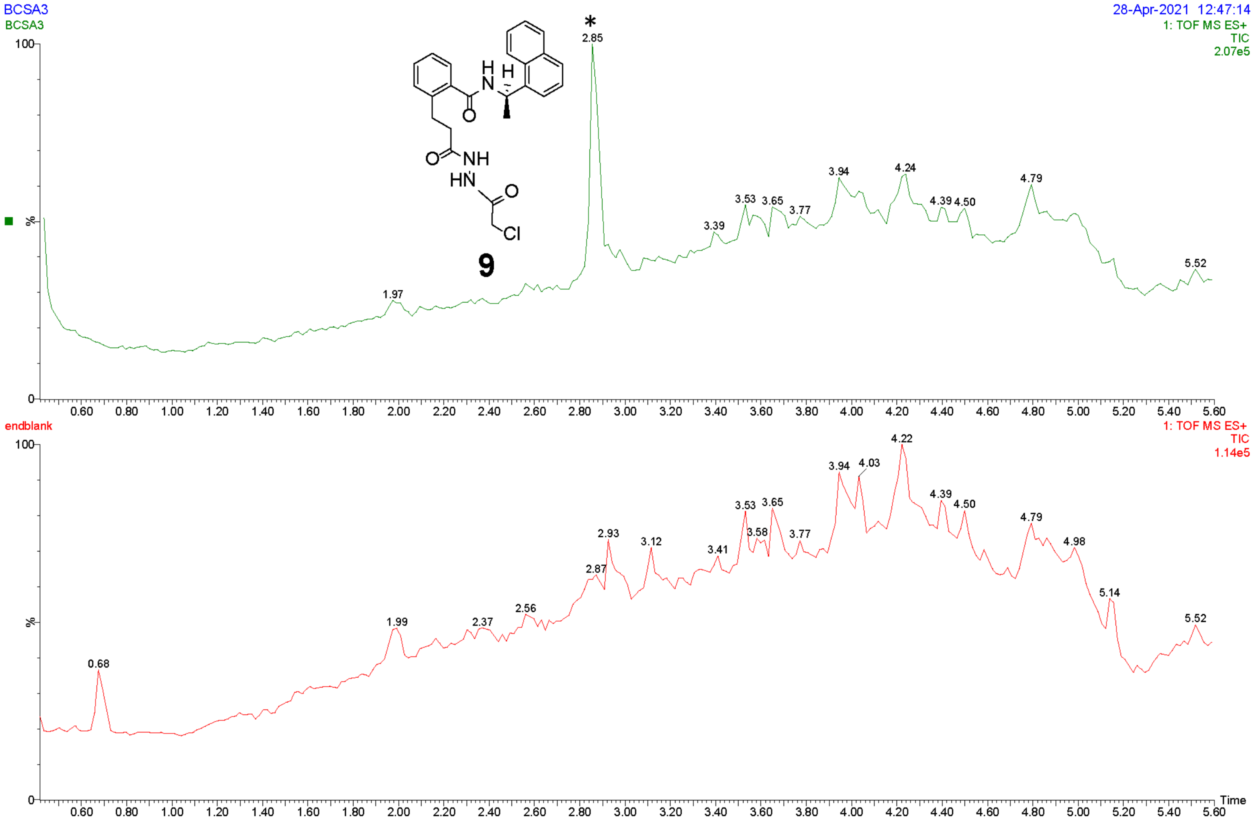

Supplementary Figure 19. LC/MS chromatogram of **9**. The top chromatogram (green) is the sample, and the bottom (red) is the background.

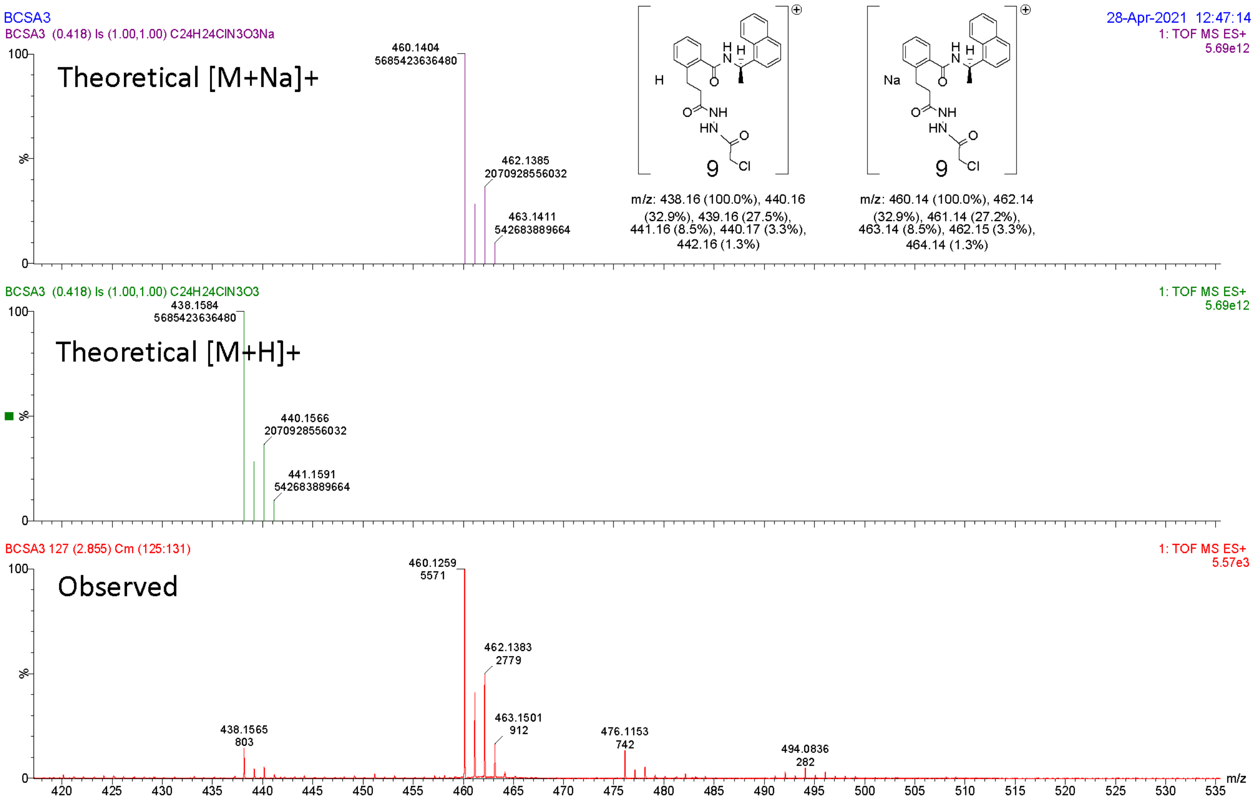

Supplementary Figure 20. HRMS of **9**.

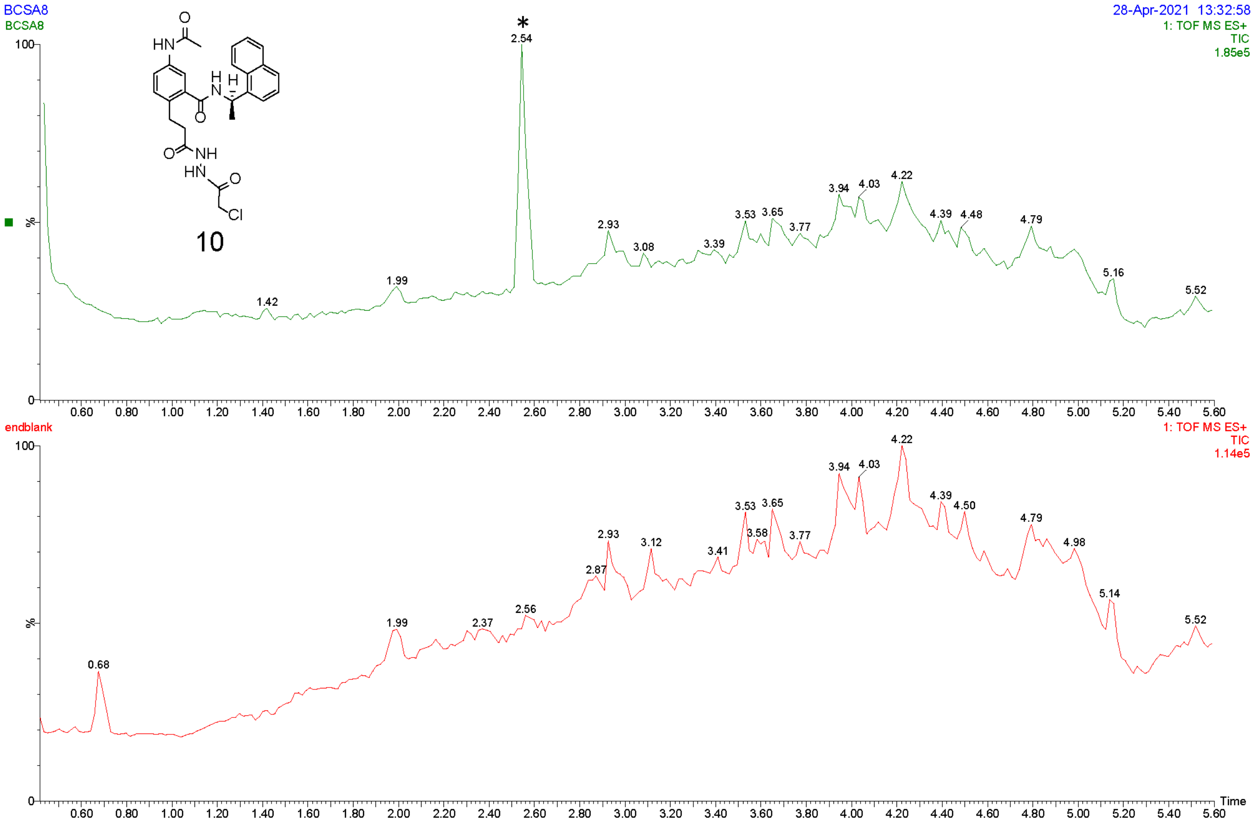

Supplementary Figure 21. LC/MS chromatogram of **10**. The top chromatogram (green) is the sample, and the bottom (red) is the background.

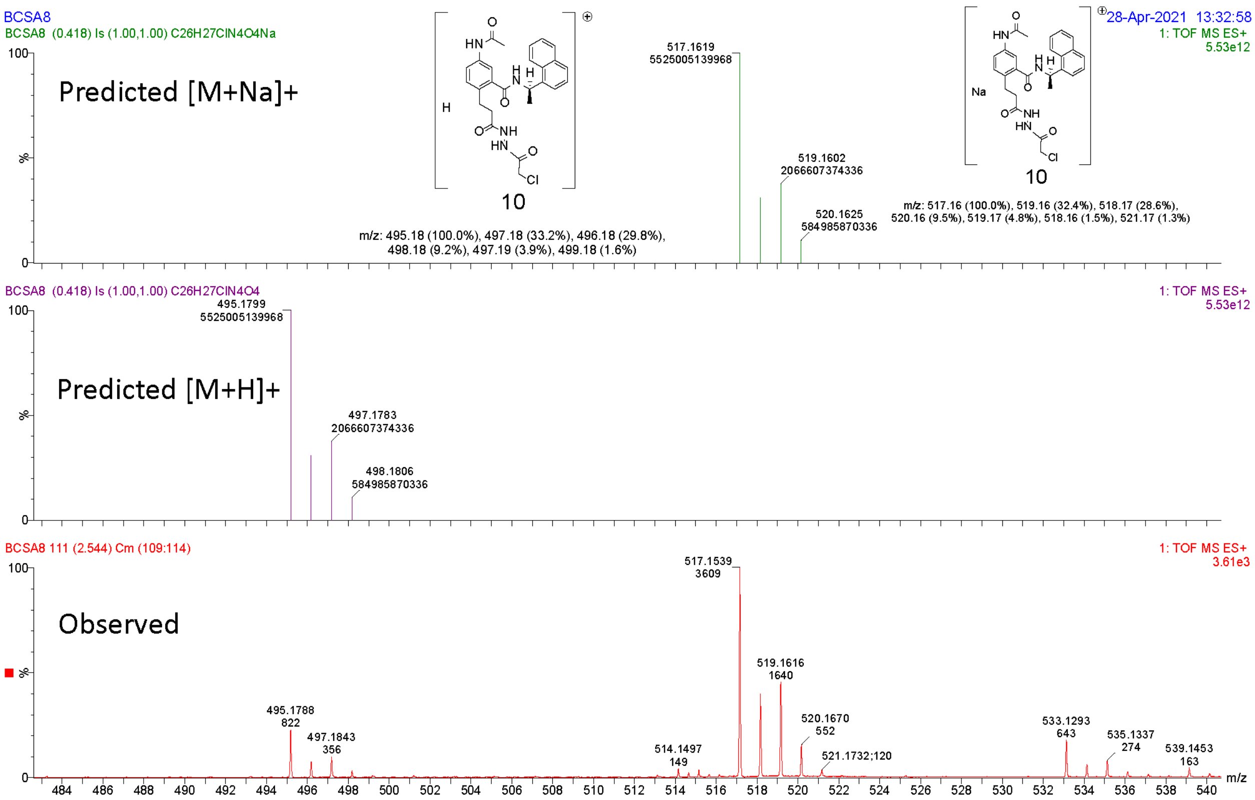

Supplementary Figure 22. HRMS of **10**.

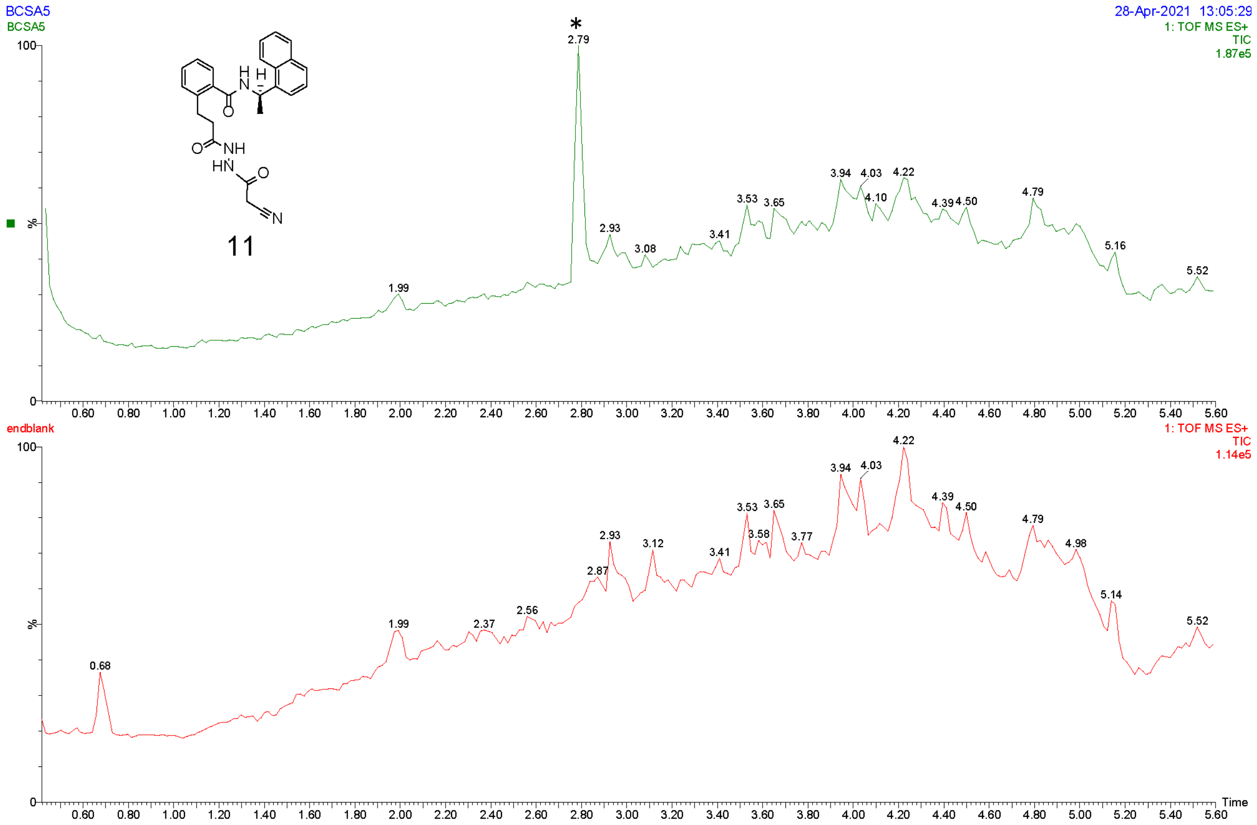

Supplementary Figure 23. LC/MS chromatogram of **11**. The top chromatogram (green) is the sample, and the bottom (red) is the background.

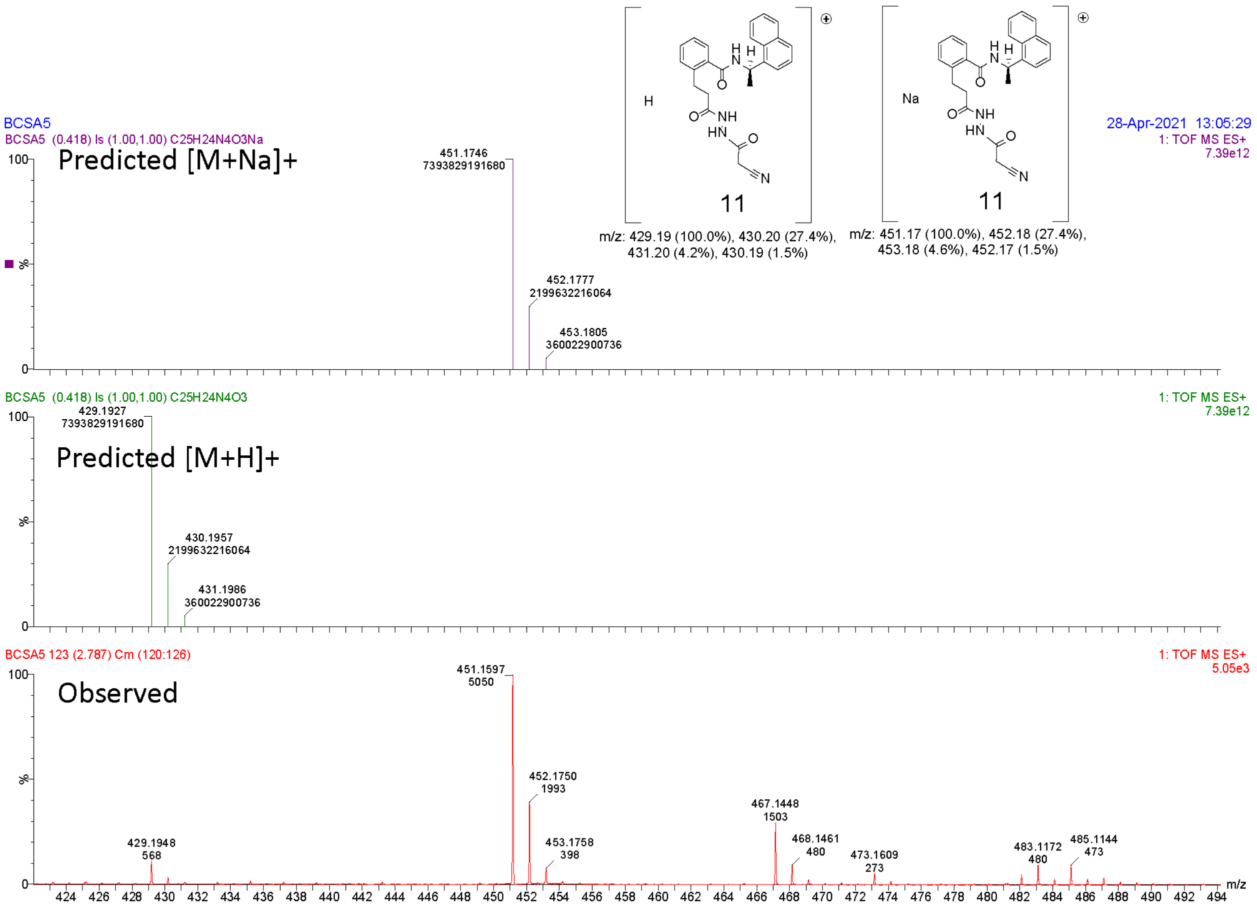

Supplementary Figure 24. HRMS of **11**.

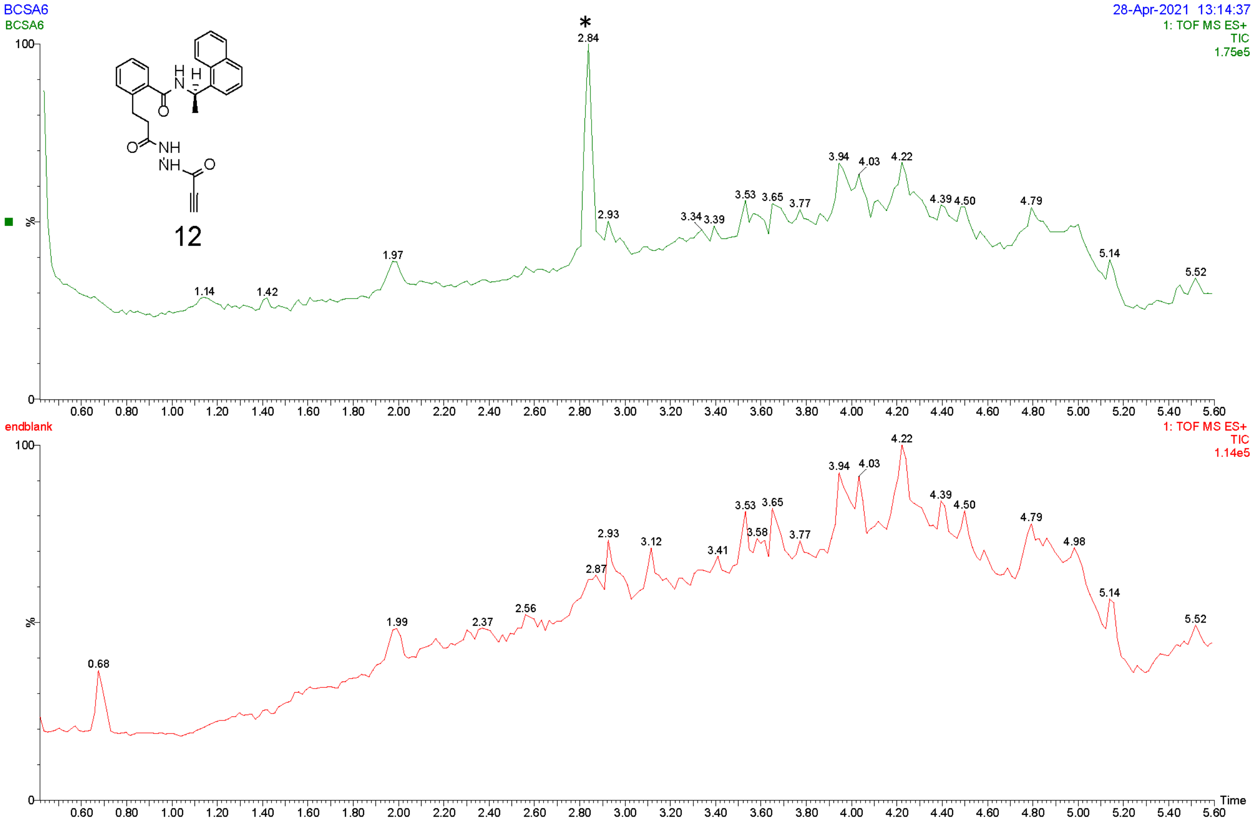

Supplementary Figure 25. LC/MS chromatogram of **12**. The top chromatogram (green) is the sample, and the bottom (red) is the background.

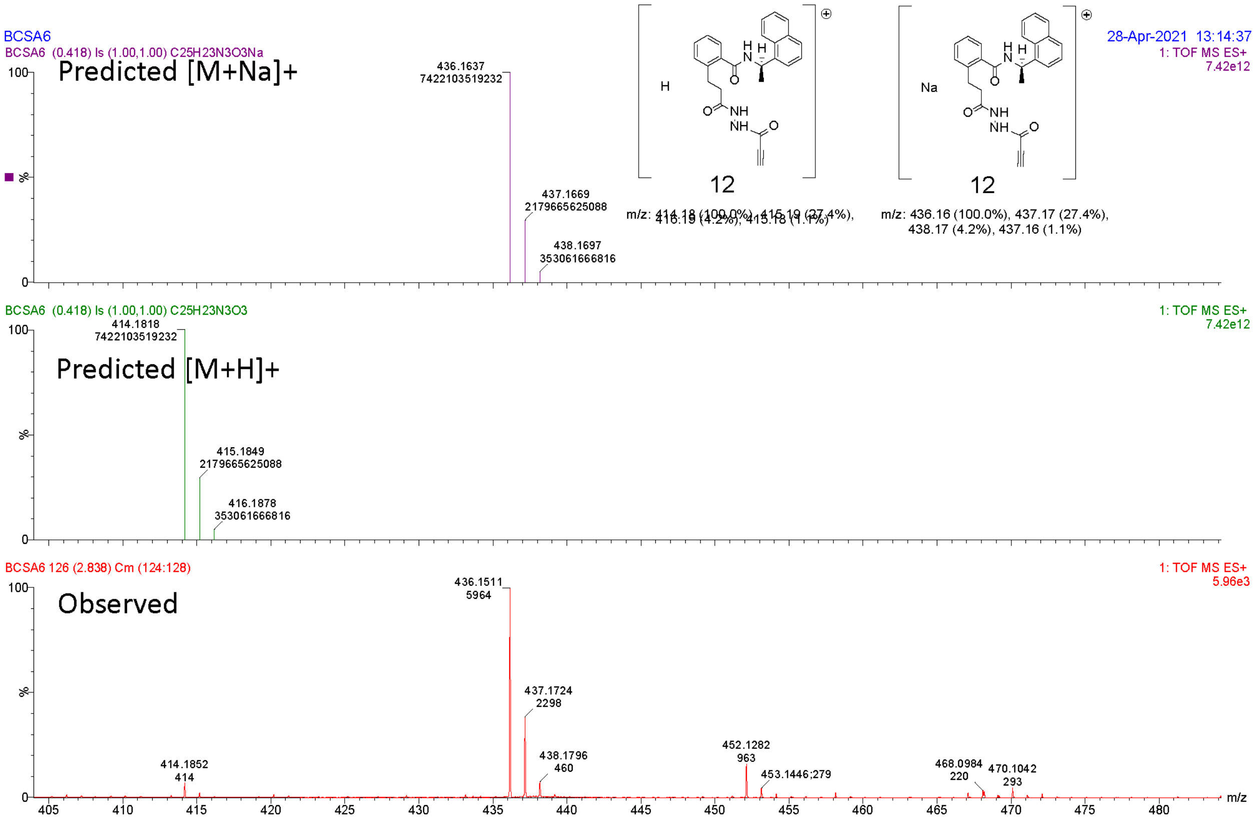

Supplementary Figure 26. HRMS of **12**.

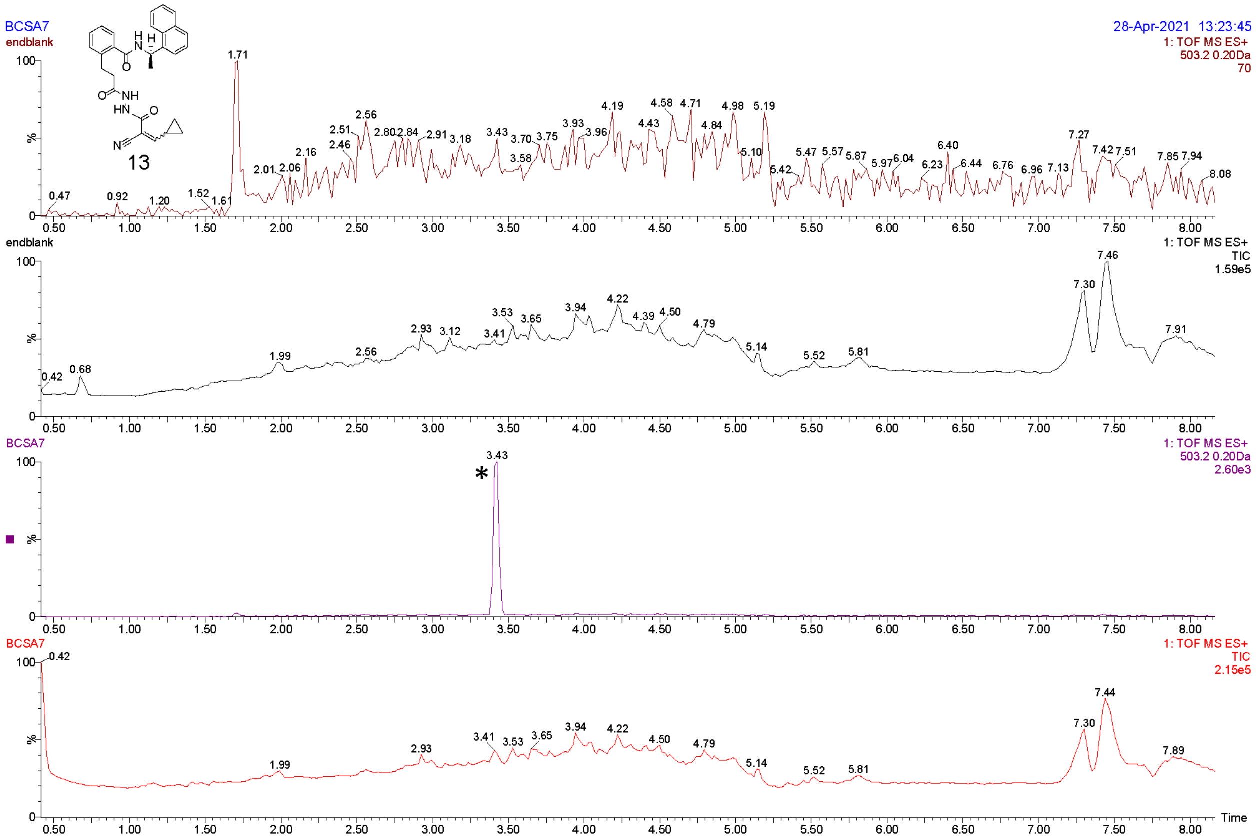

Supplementary Figure 27. LC/MS chromatogram of **13**. The top two chromatograms (burgundy and black) are the backgrounds and the bottom two (purple and red) are samples.

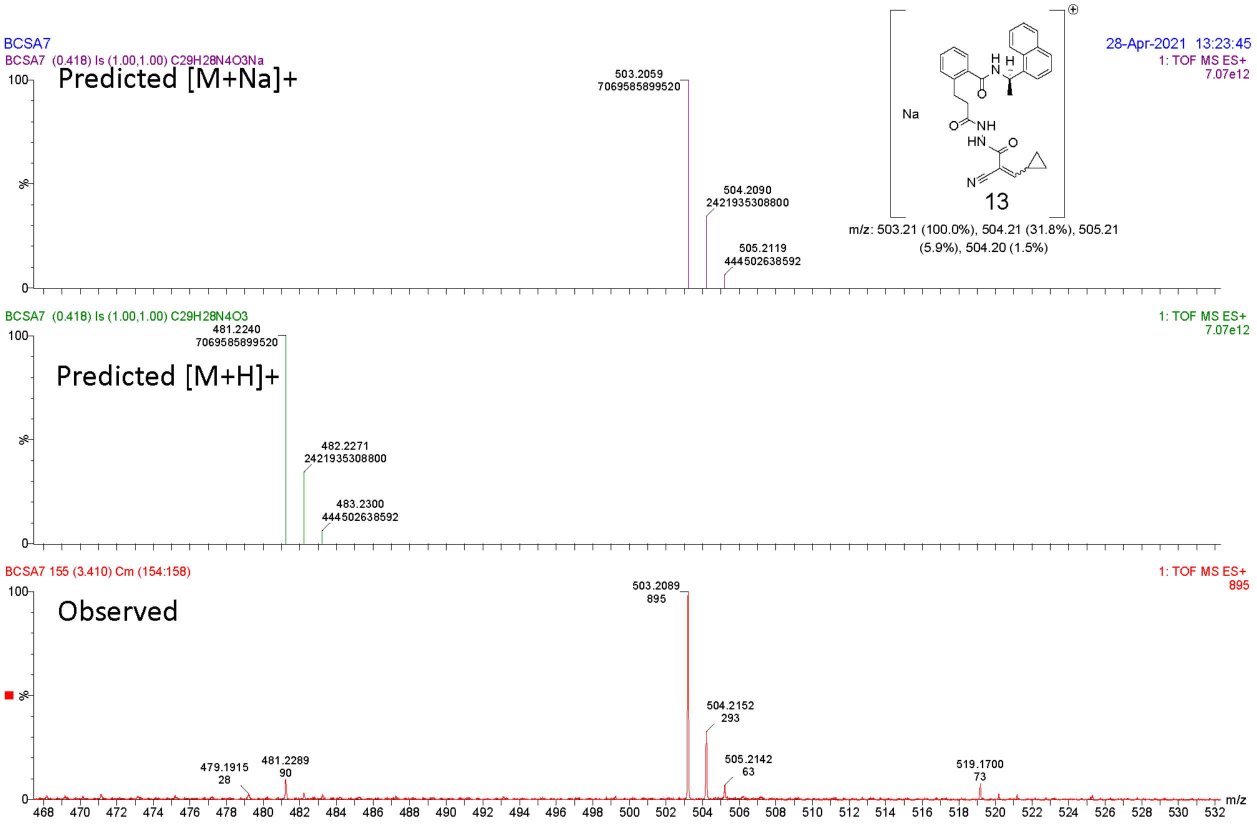

Supplementary Figure 28. HRMS of **13**.

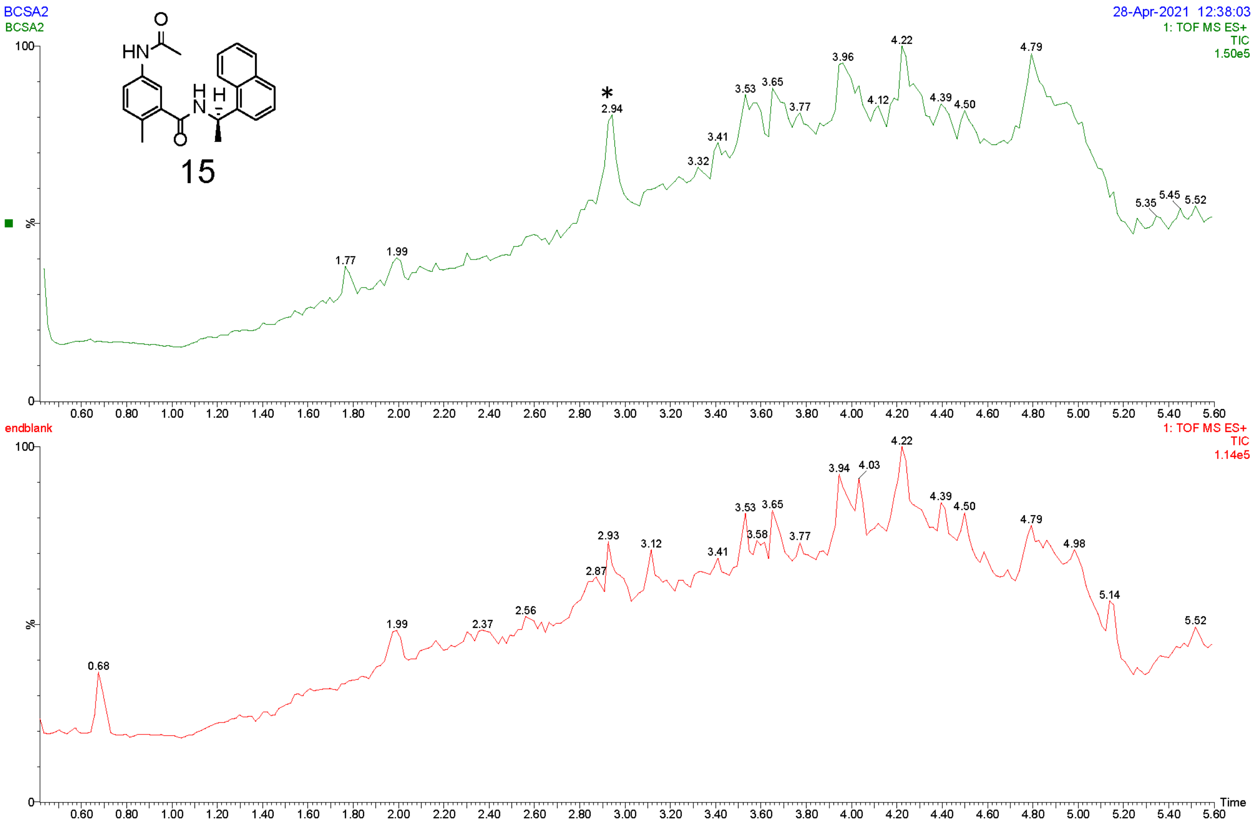

Supplementary Figure 29. LC/MS chromatogram of **15**. The top chromatogram (green) is the sample, and the bottom (red) is the background.

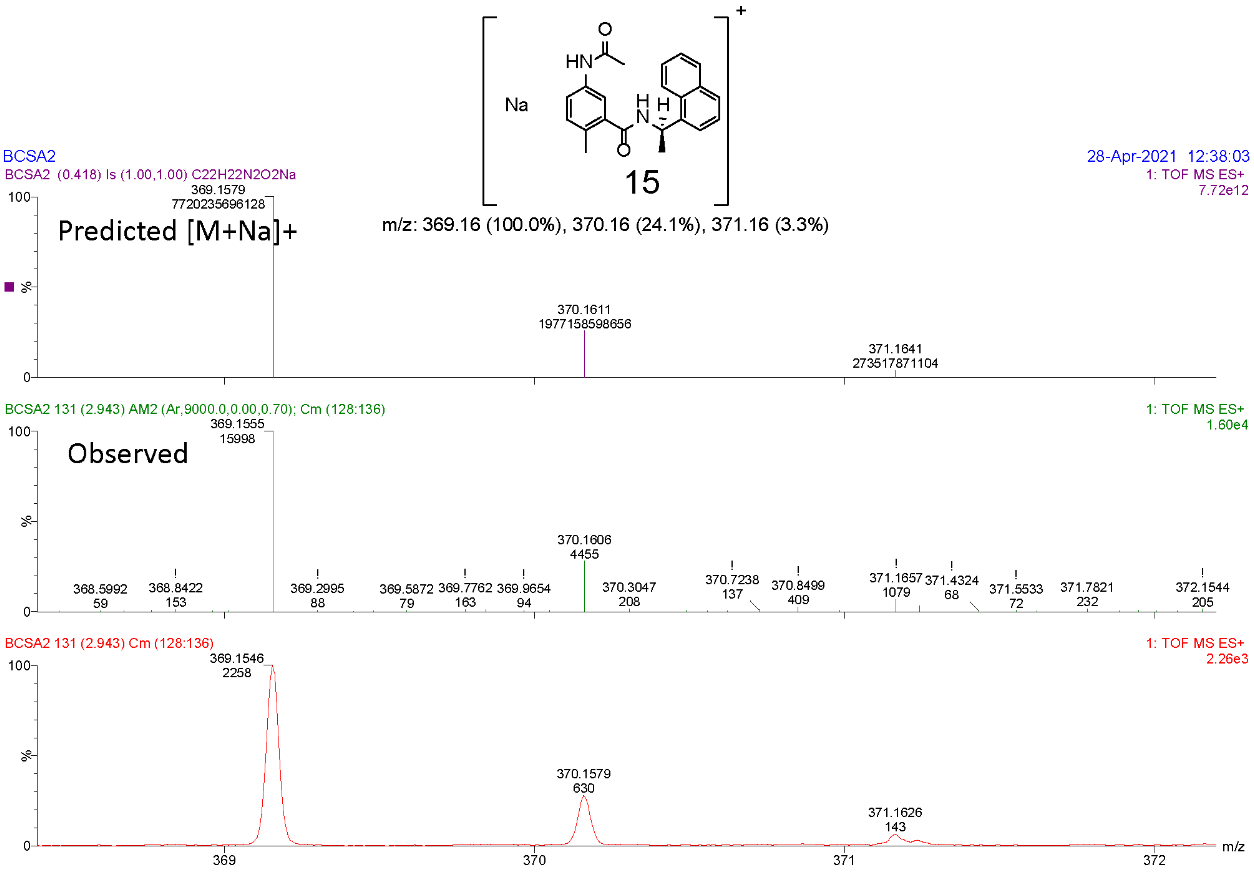

Supplementary Figure 30 HRMS of **15**.

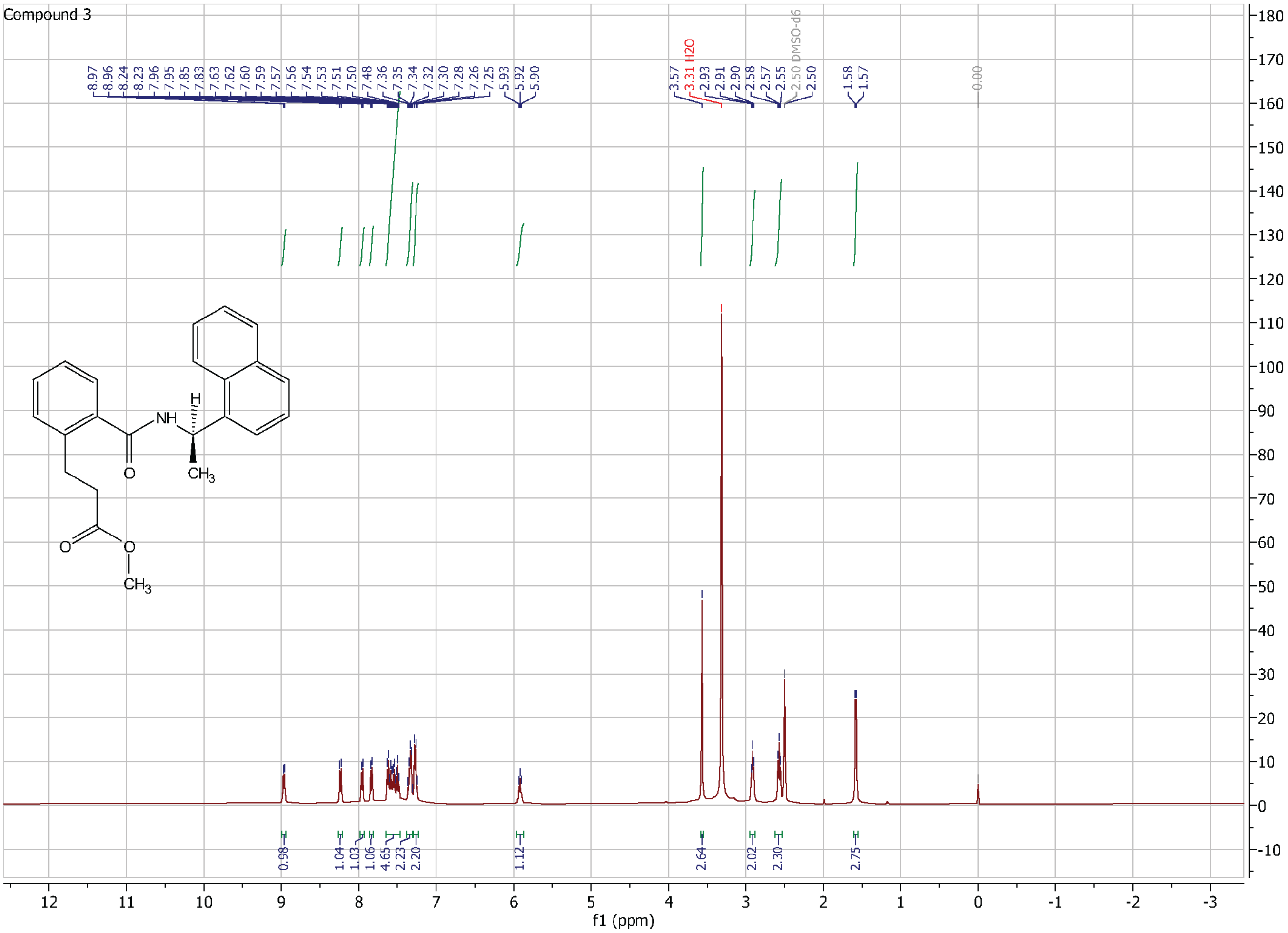

Supplementary Figure 31. ^{1}H NMR spectrum of **3**.

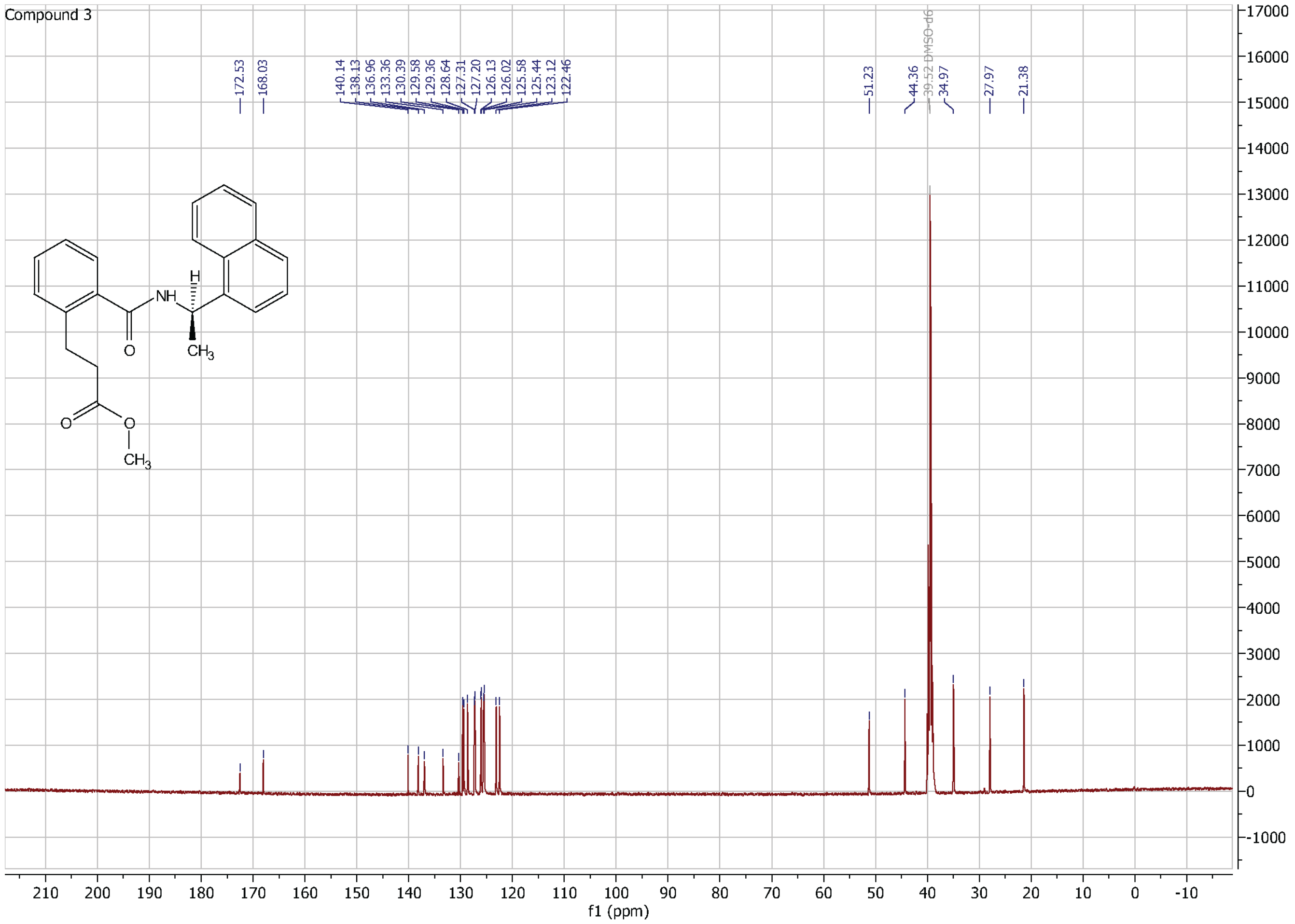

Supplementary Figure 32. ^{13}C NMR spectrum of **3**.

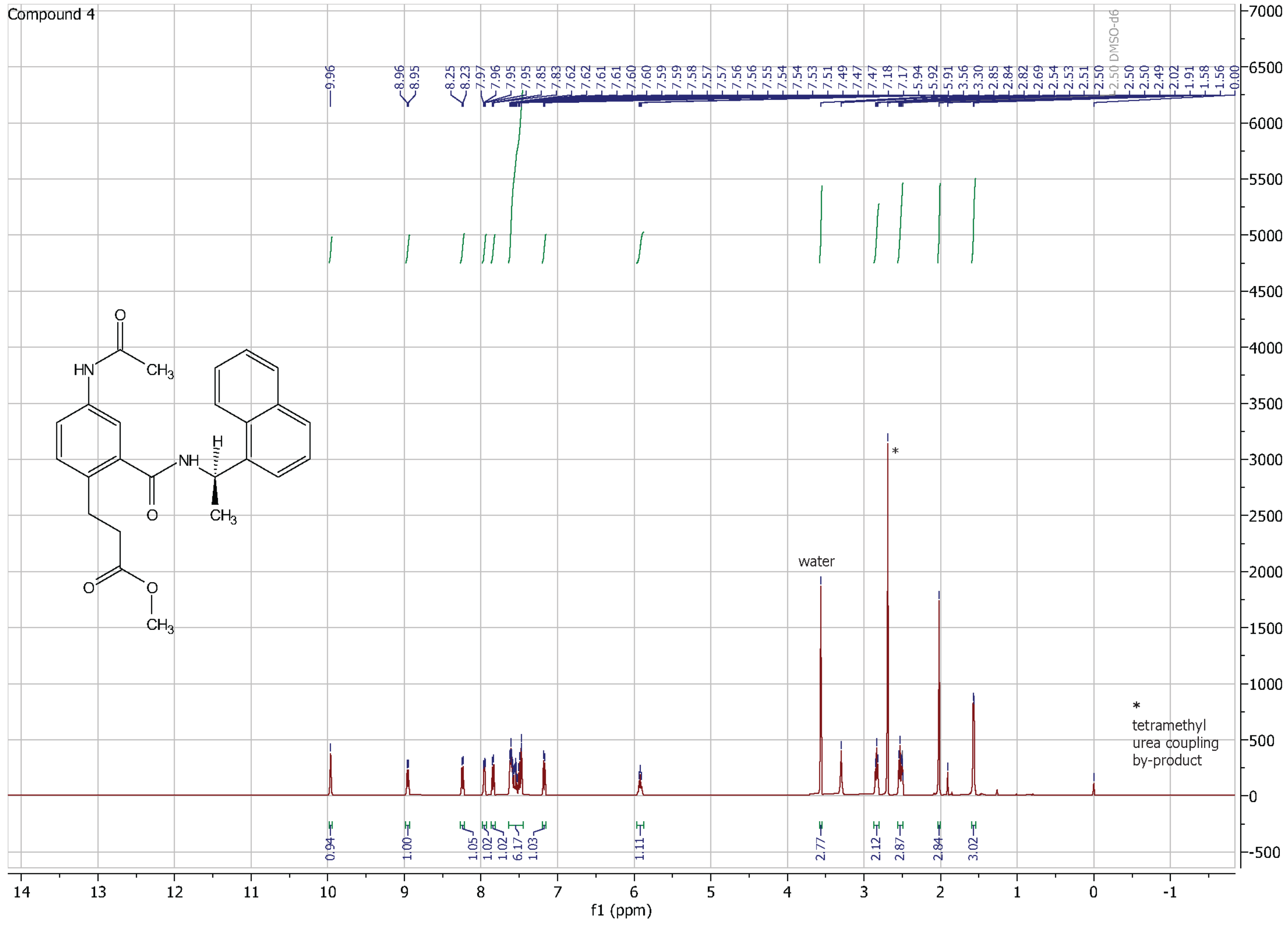

Supplementary Figure 33. ^{1}H NMR spectrum of **4**.

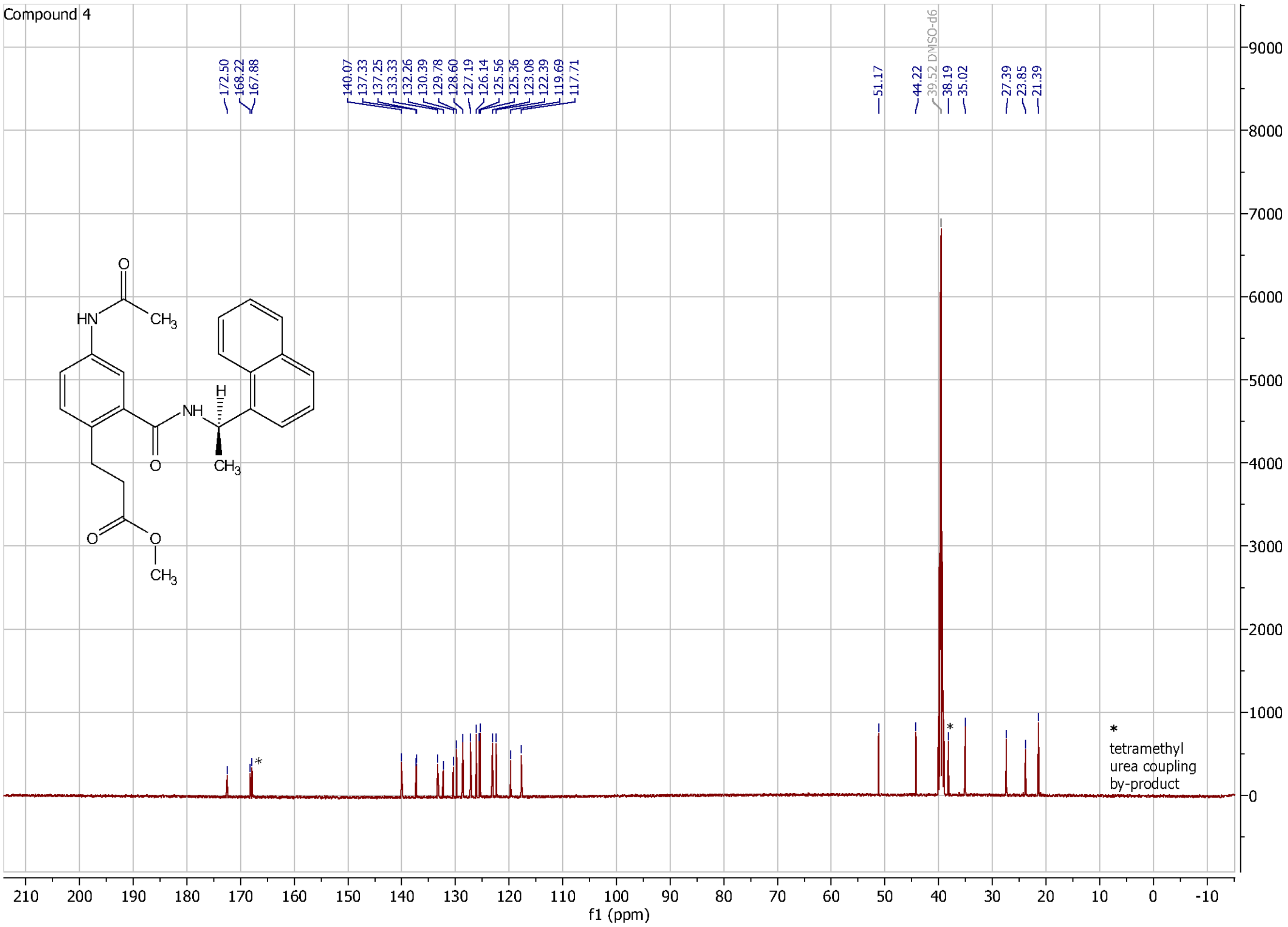

Supplementary Figure 34. ^{13}C NMR spectrum of **4**.

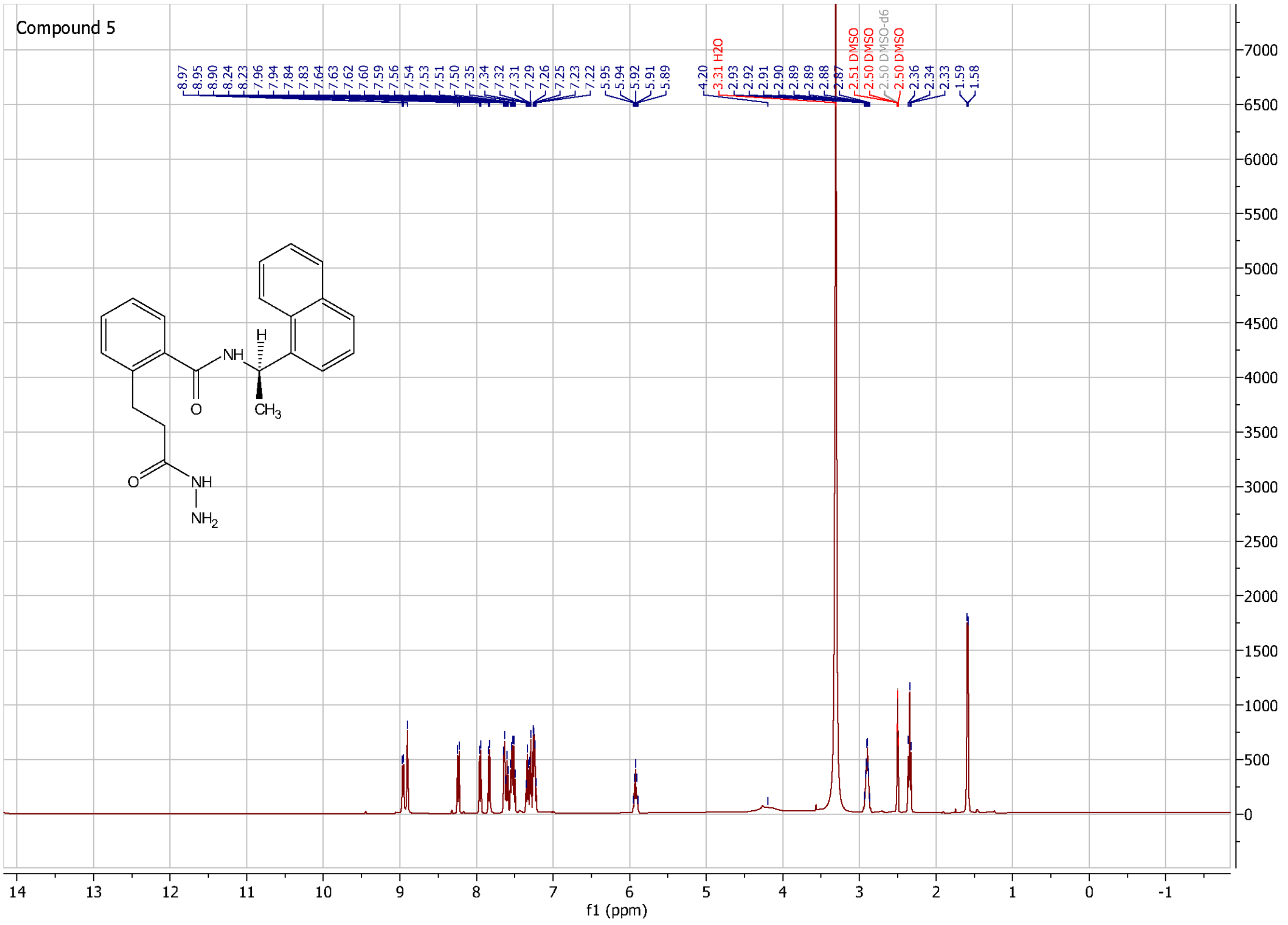

Supplementary Figure 35. ^{1}H NMR spectrum of **5**.

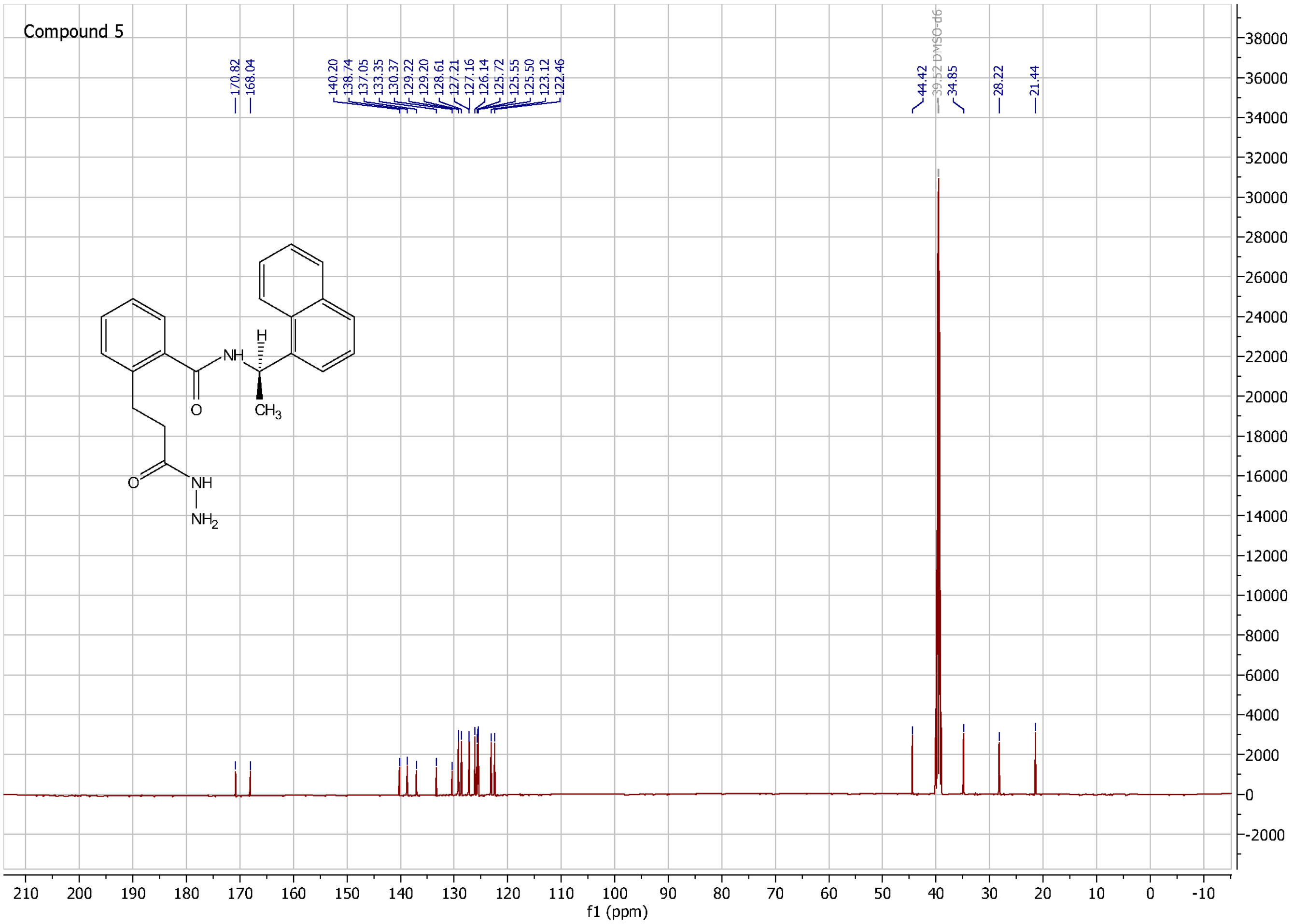

Supplementary Figure 36. ^{13}C NMR spectrum of **5**.

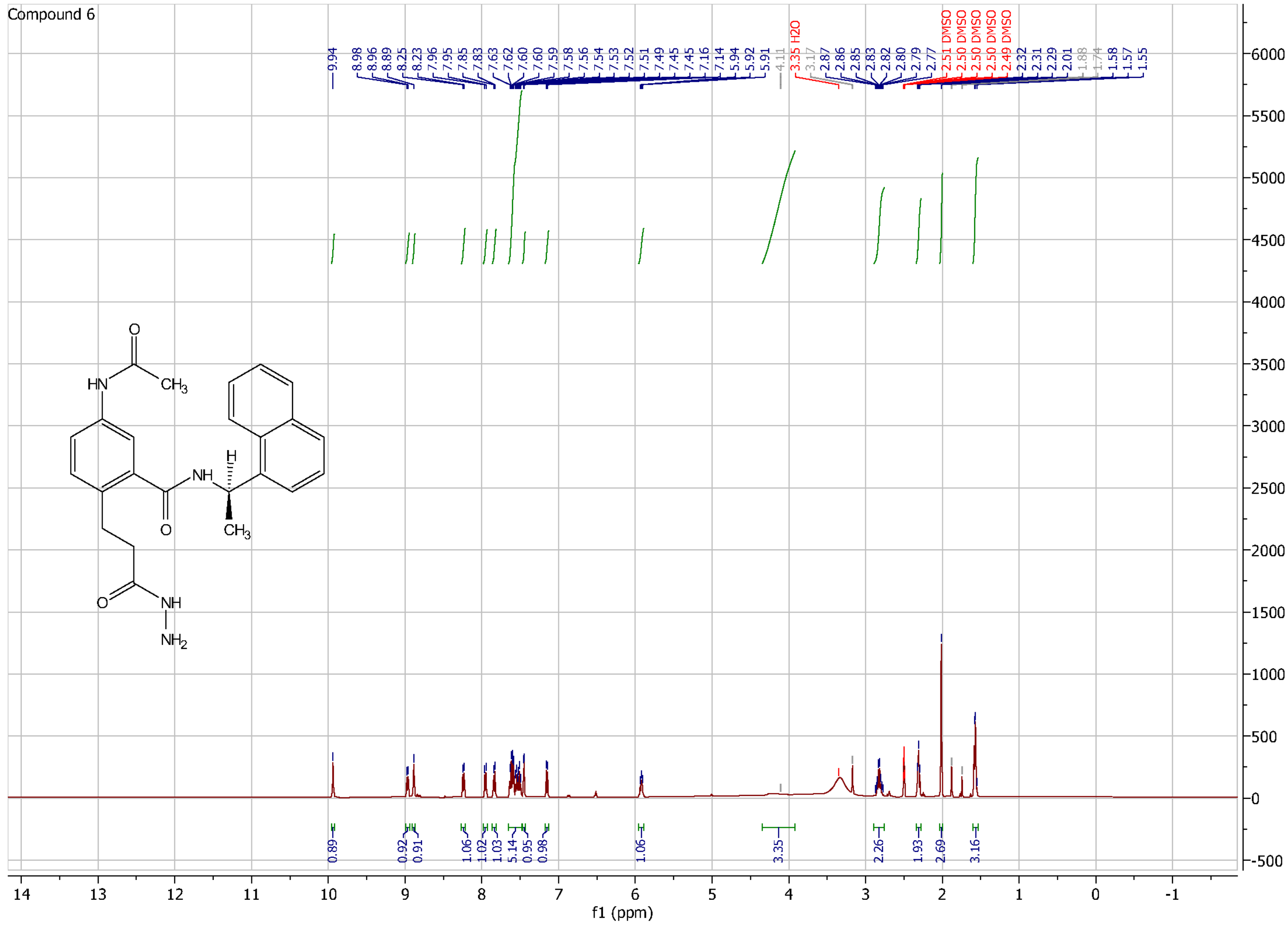

Supplementary Figure 37. ^{1}H NMR spectrum of **6**.

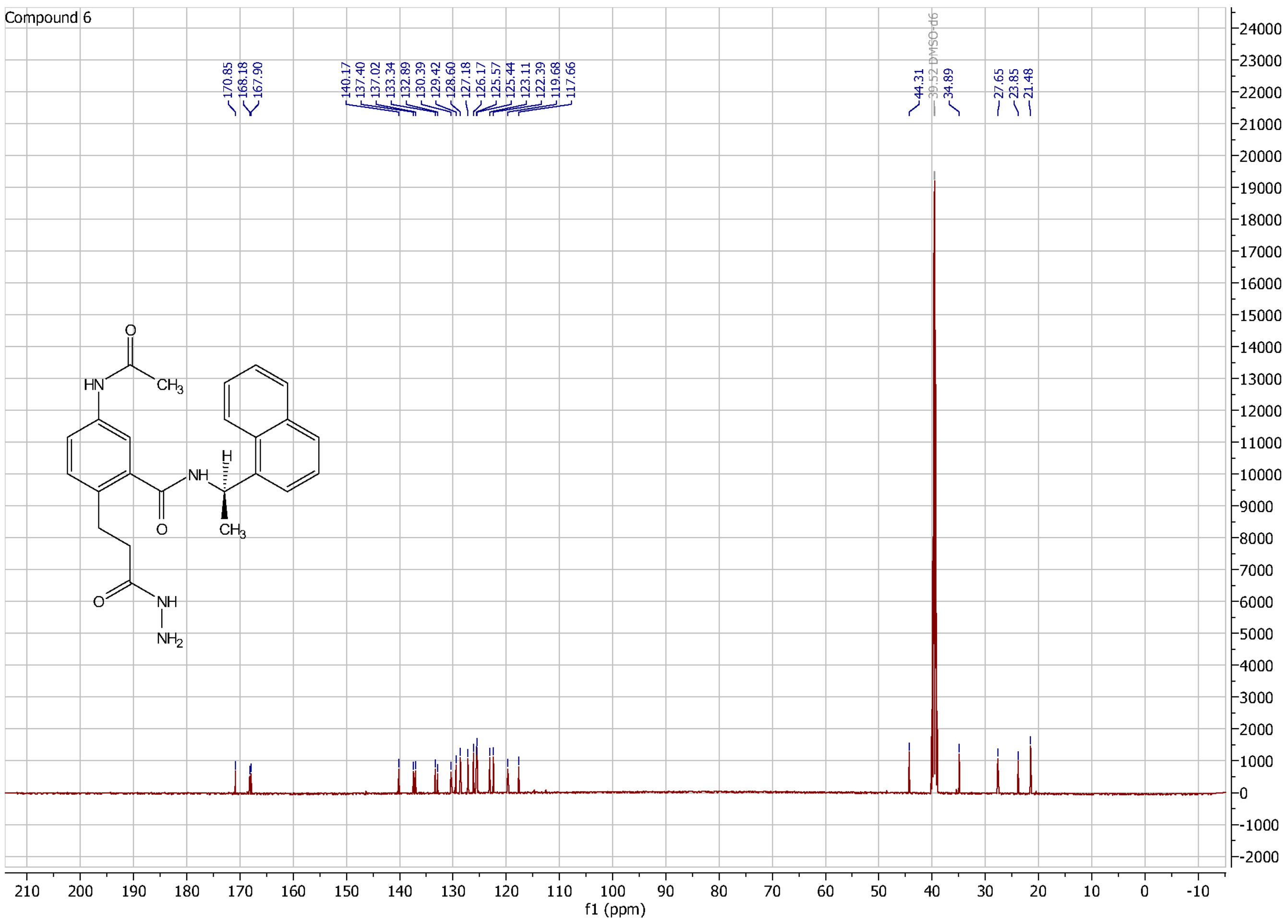

Supplementary Figure 38. ^{13}C NMR spectrum of **6.**

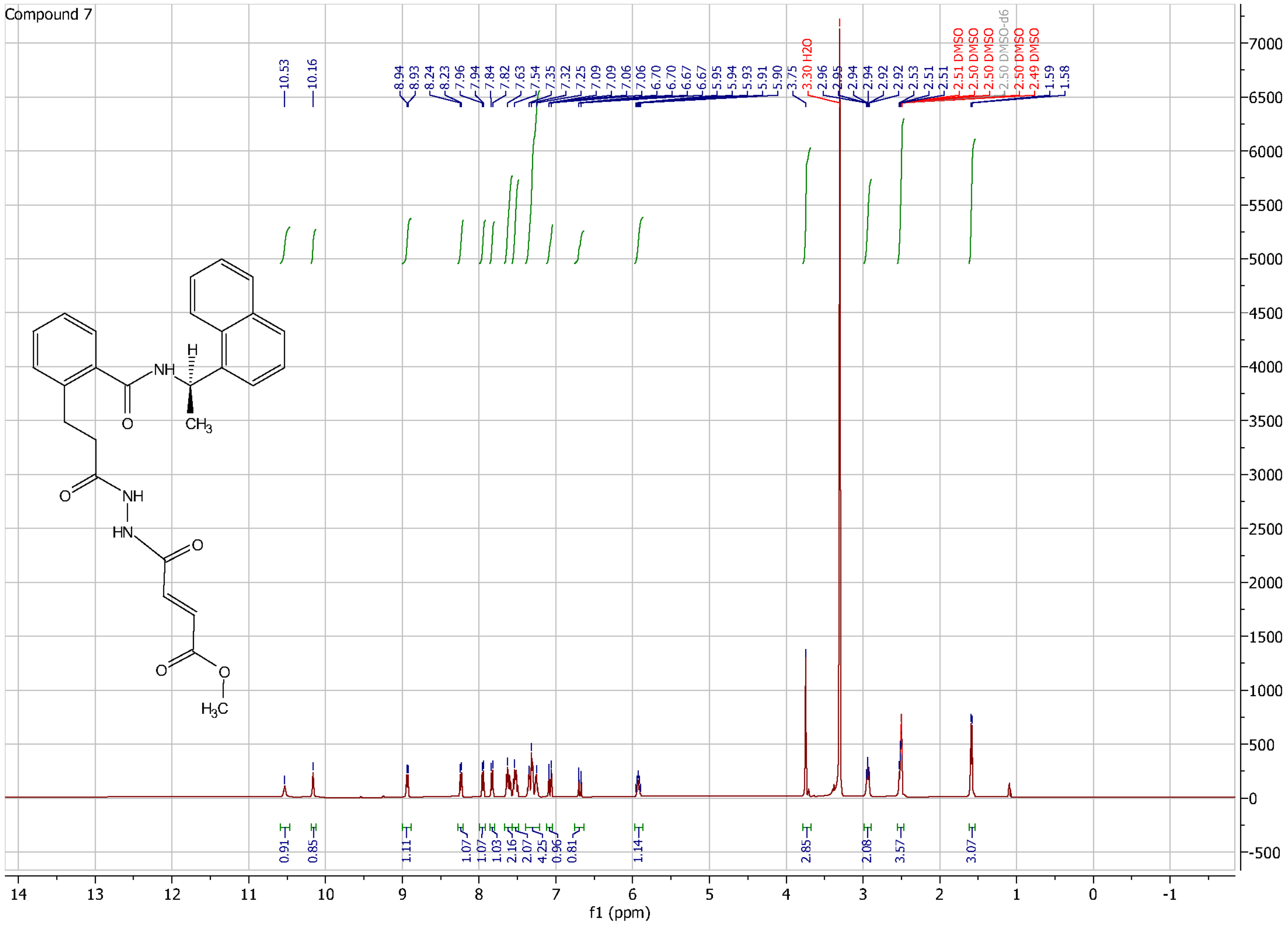

Supplementary Figure 39. ^{1}H NMR spectrum of **7.**

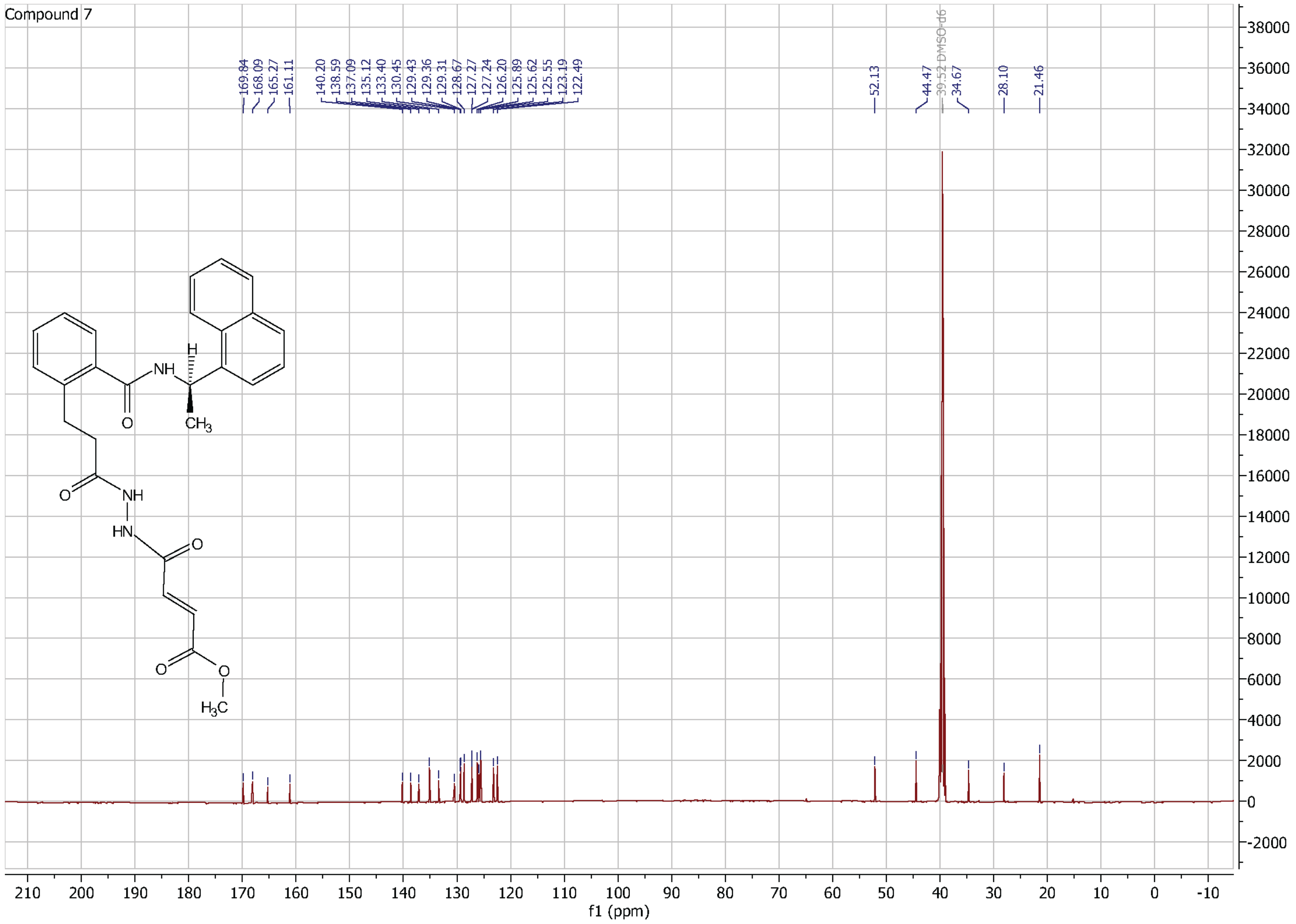

Supplementary Figure 40. ^{13}C NMR spectrum of **7**

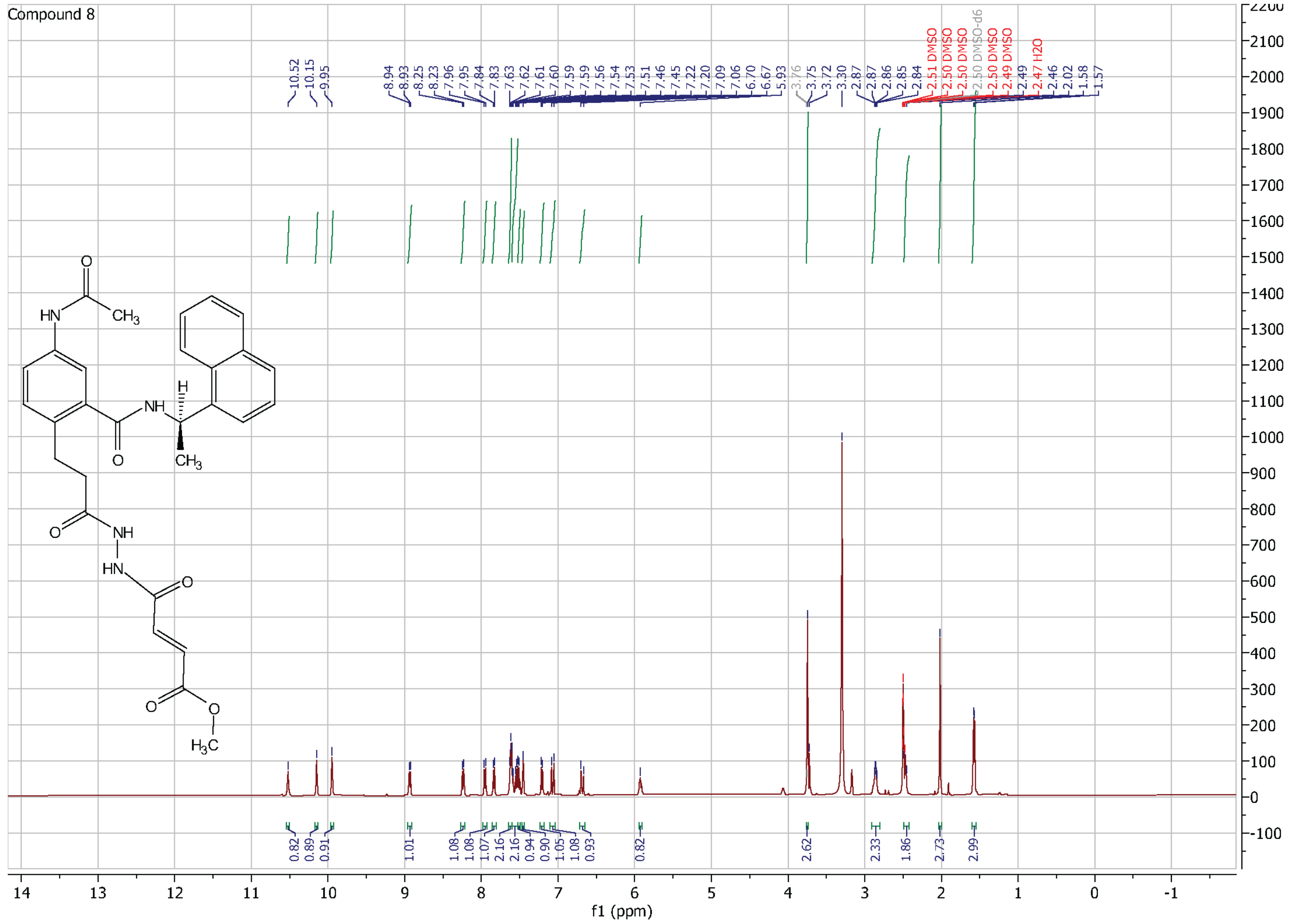

Supplementary Figure 41. ^{1}H NMR spectrum of **8**.

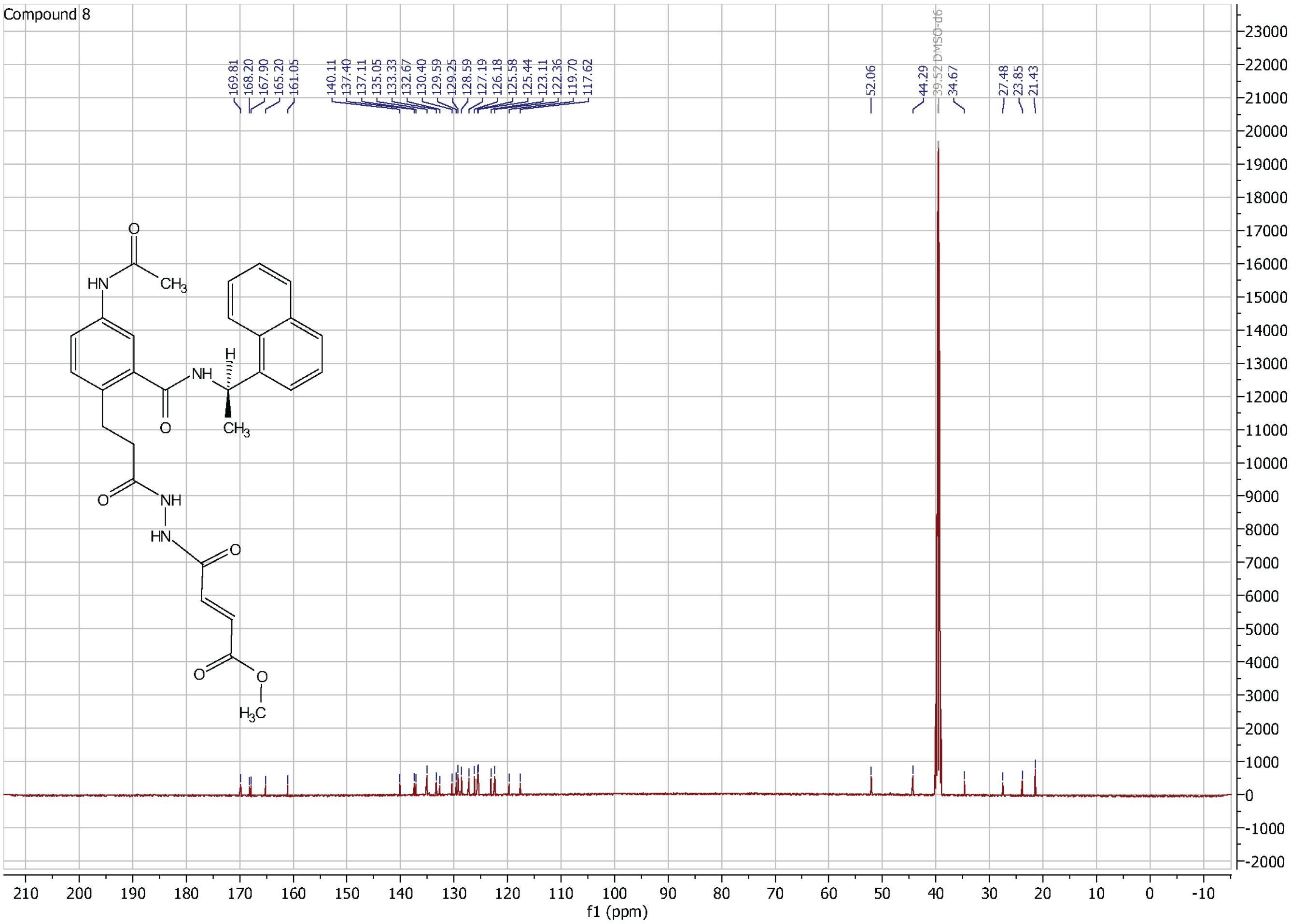

Supplementary Figure 42. ^{13}C NMR spectrum of **8**.

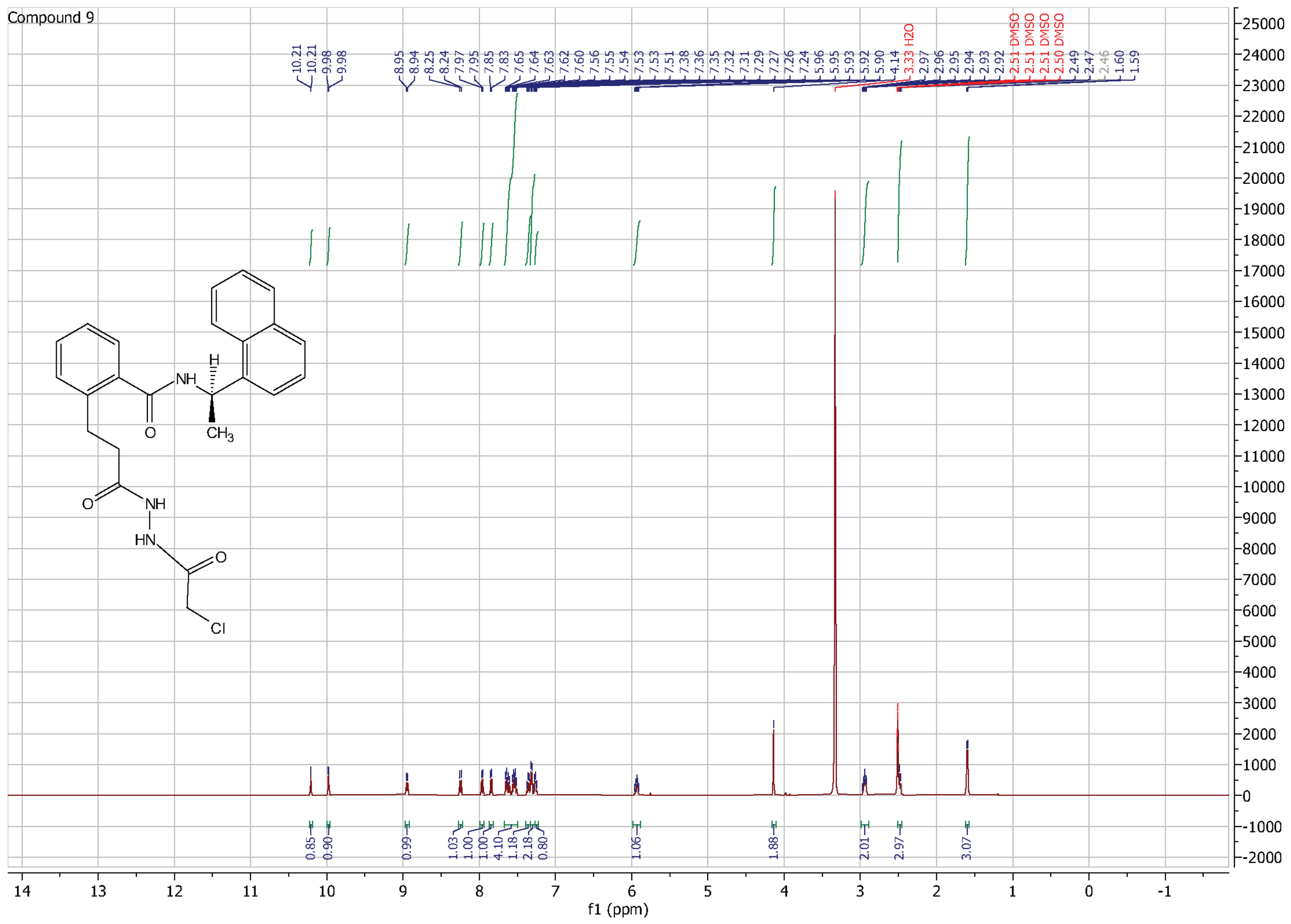

Supplementary Figure 43. ^{1}H NMR spectrum of **9**.

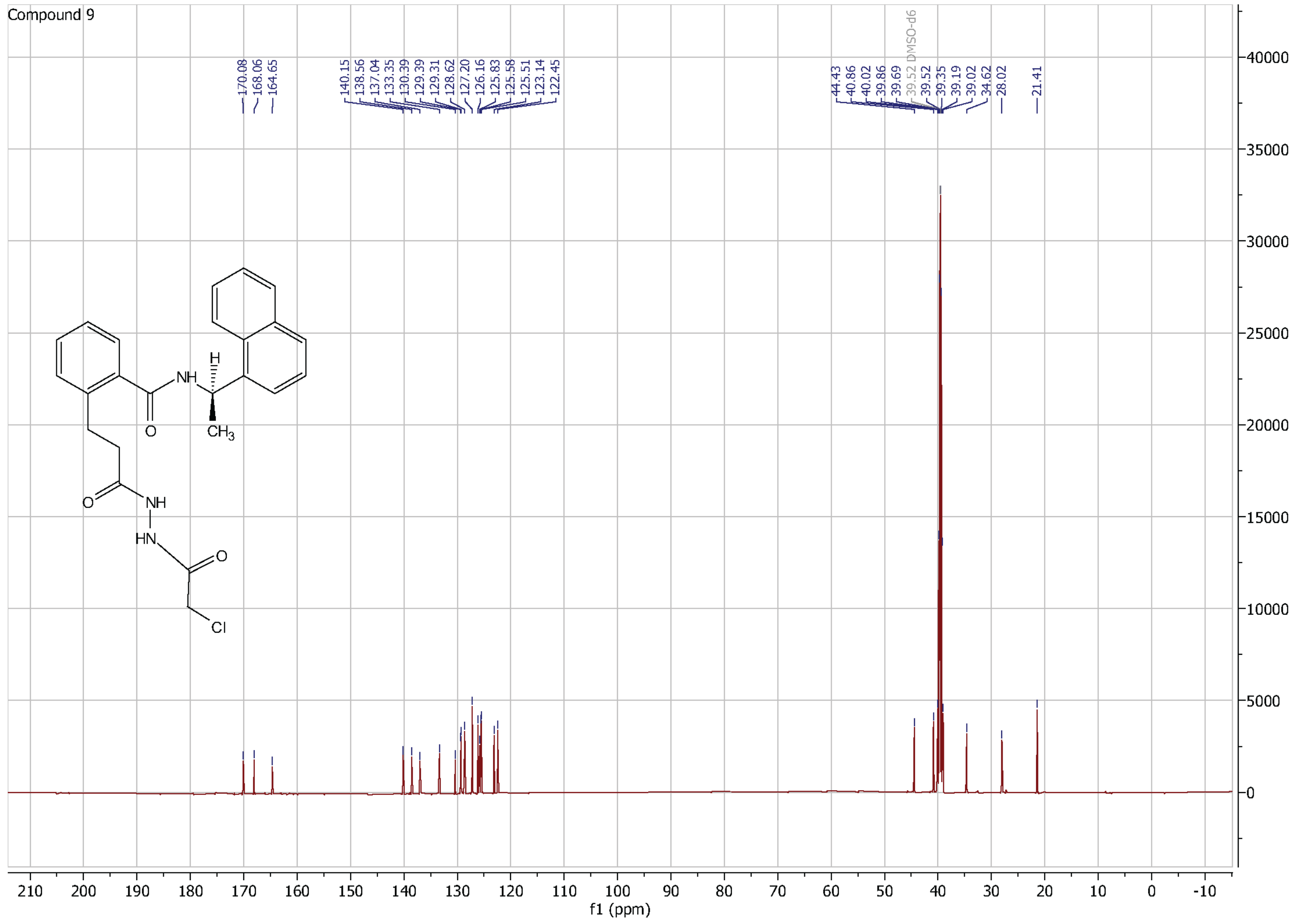

Supplementary Figure 44. ^{13}C NMR spectrum of **9**.

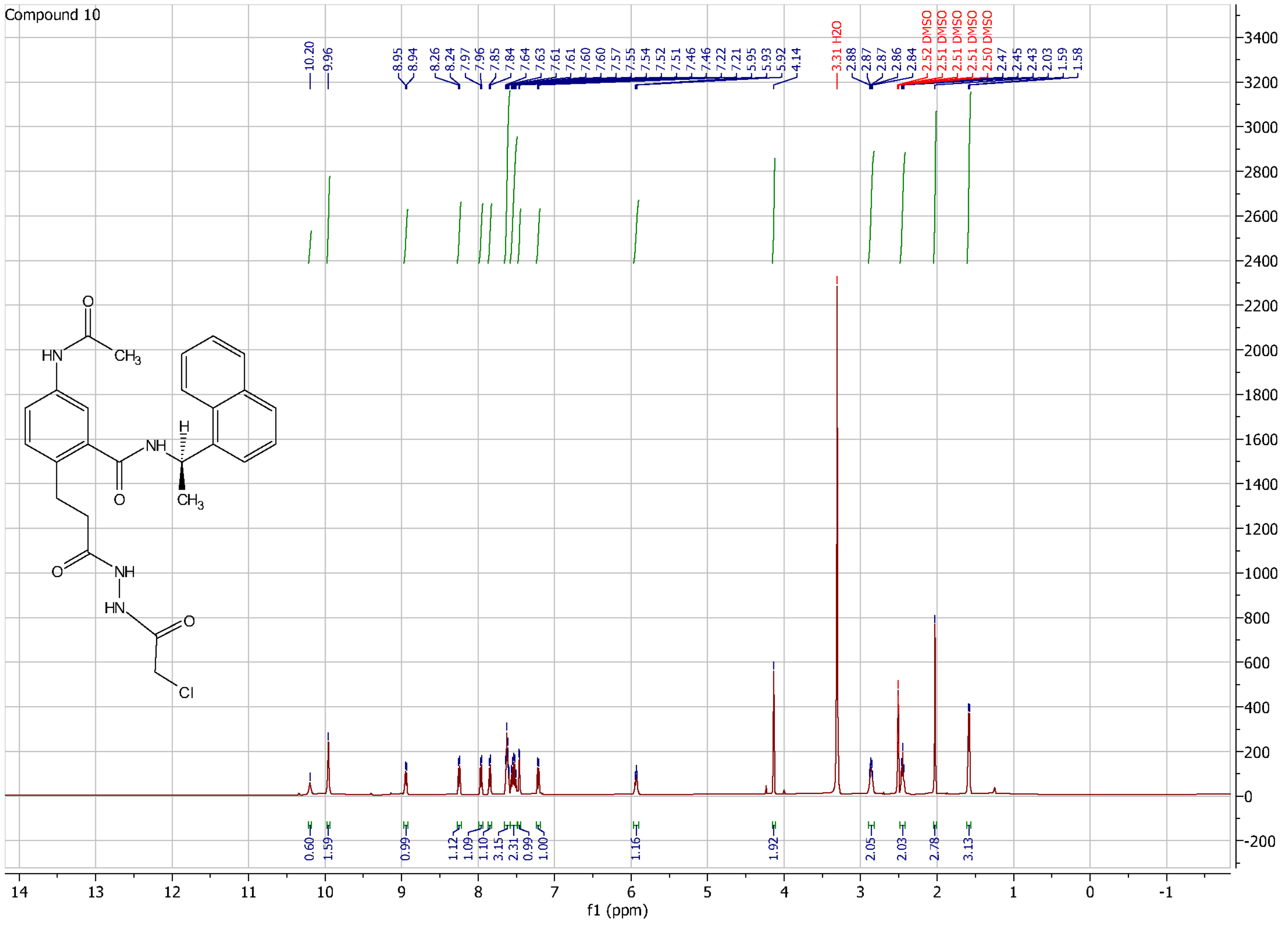

Supplementary Figure 45. ^{1}H NMR spectrum of **10**.

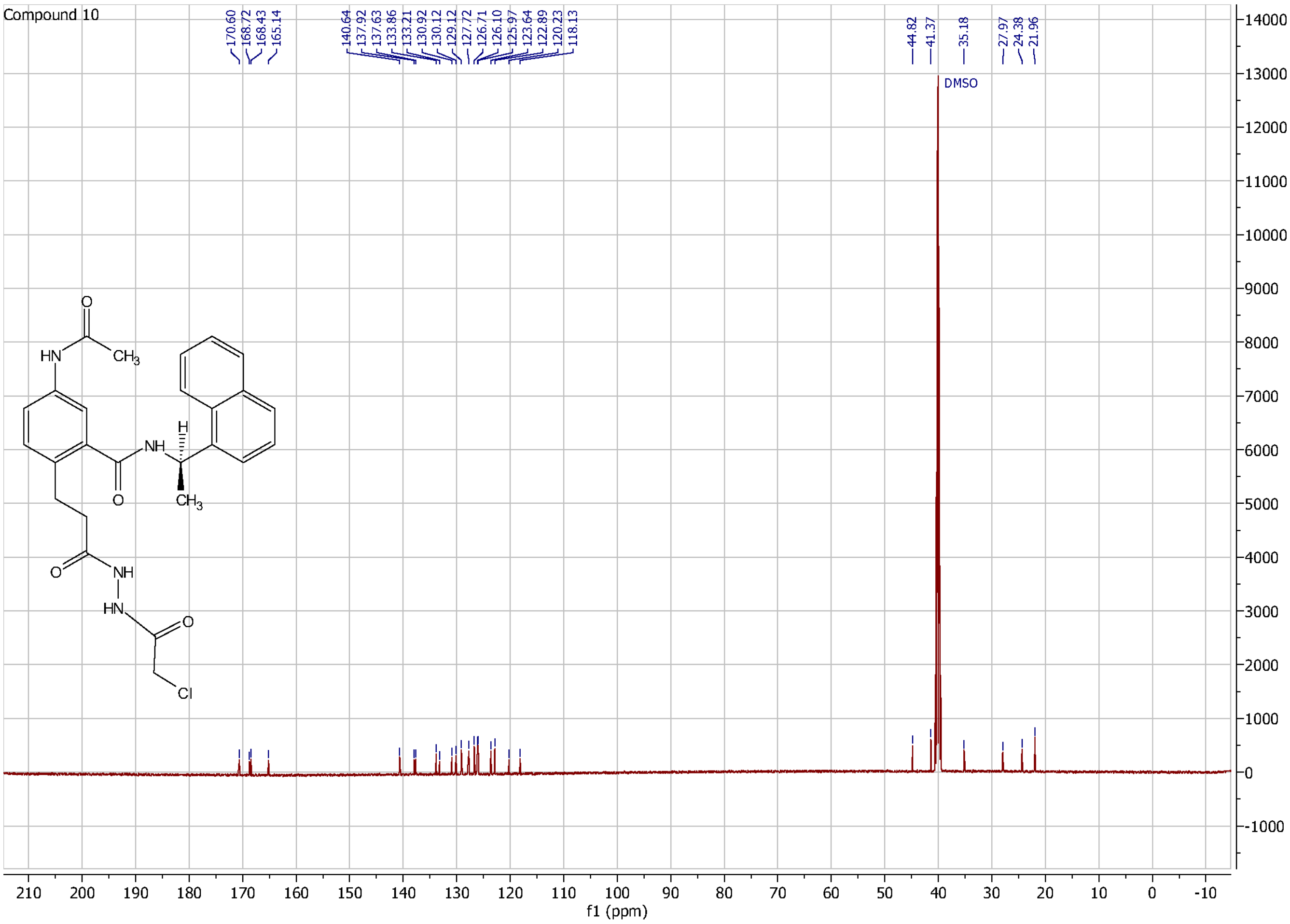

Supplementary Figure 46. ^{13}C NMR spectrum of **10**.

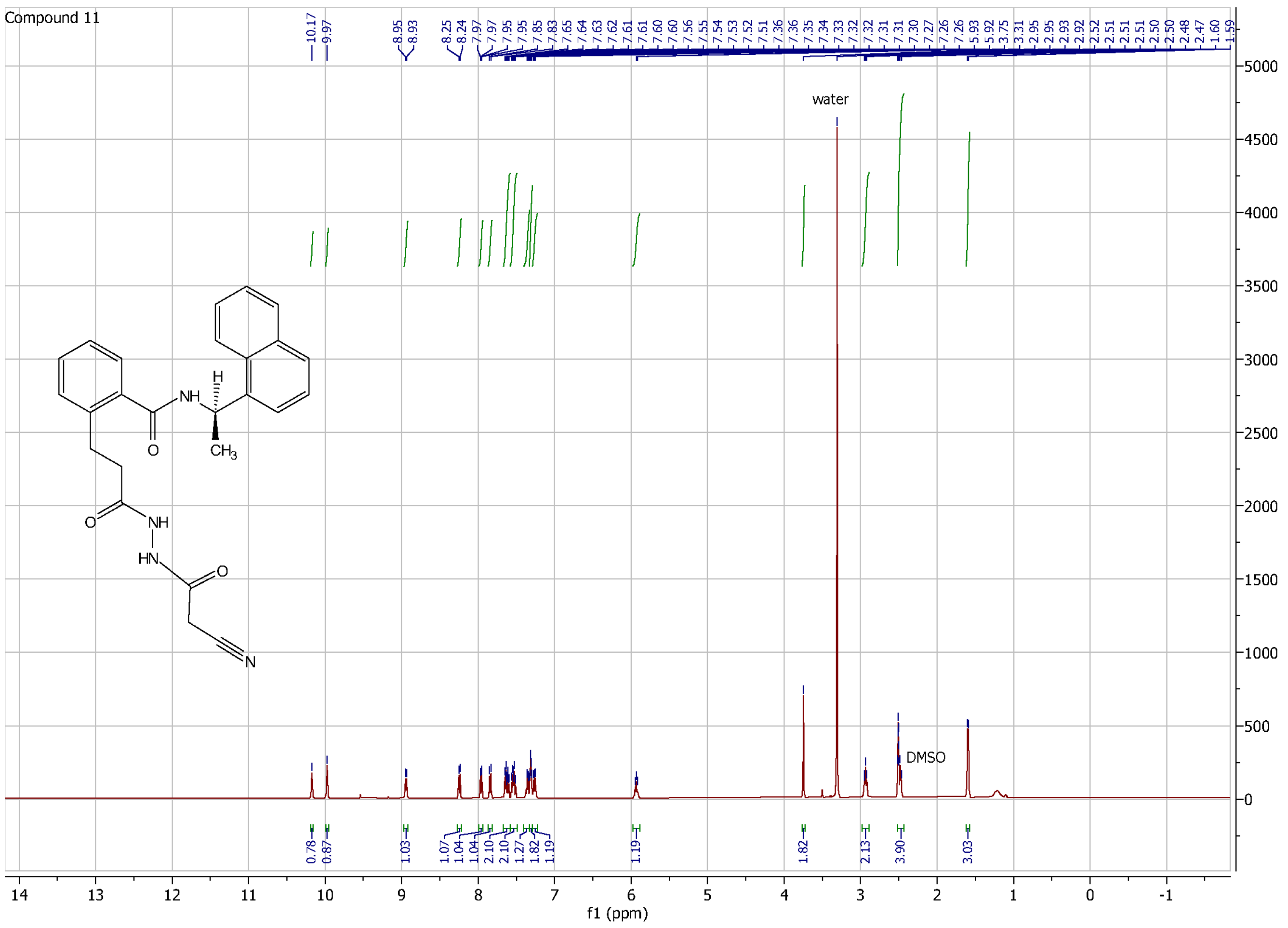

Supplementary Figure 47. ^{1}H NMR spectrum of **11**.

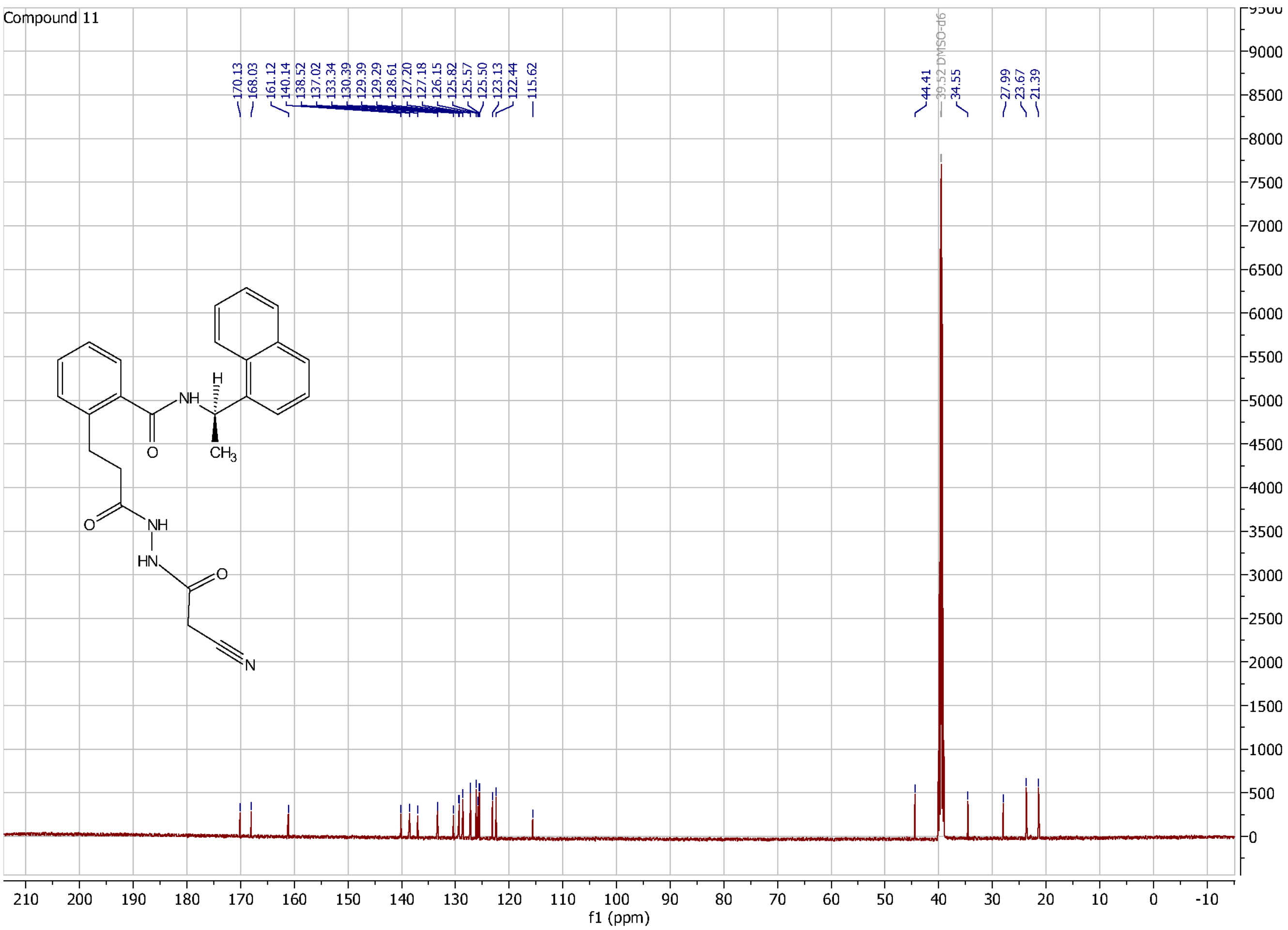

Supplementary Figure 48. ^{13}C NMR spectrum of **11**.

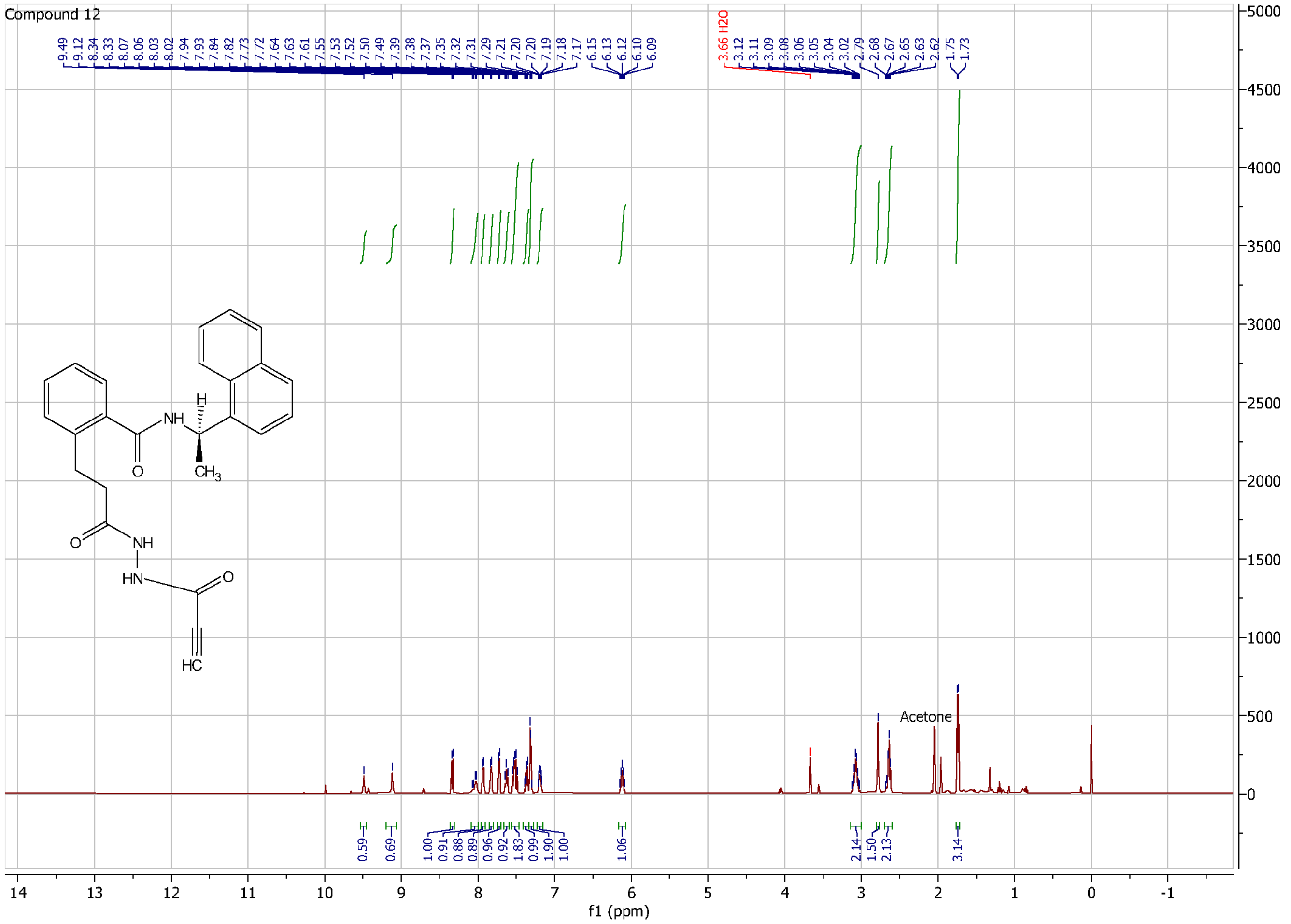

Supplementary Figure 49. ^{1}H NMR spectrum of **12**.

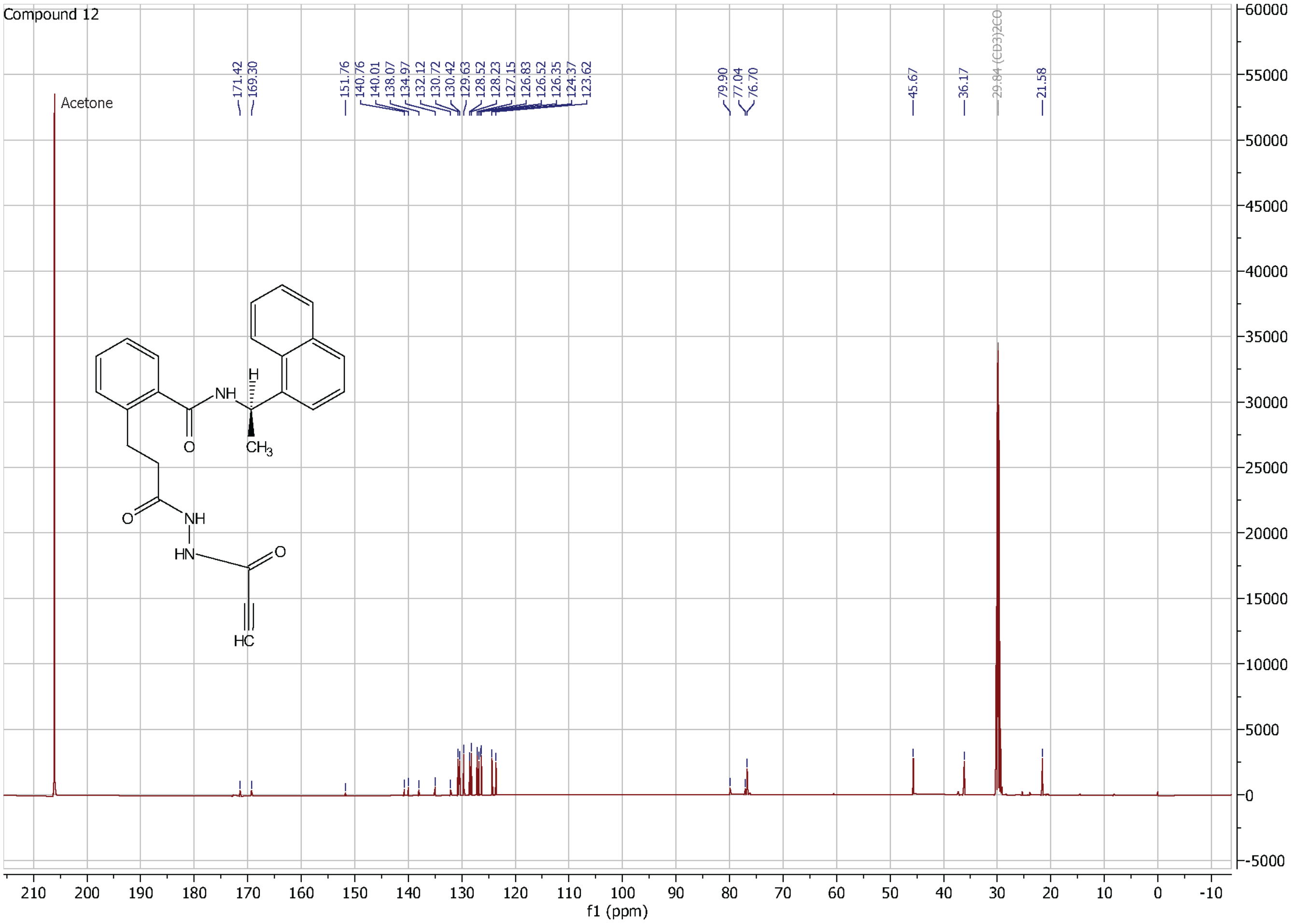

Supplementary Figure 50. ^{13}C NMR spectrum of **12**.

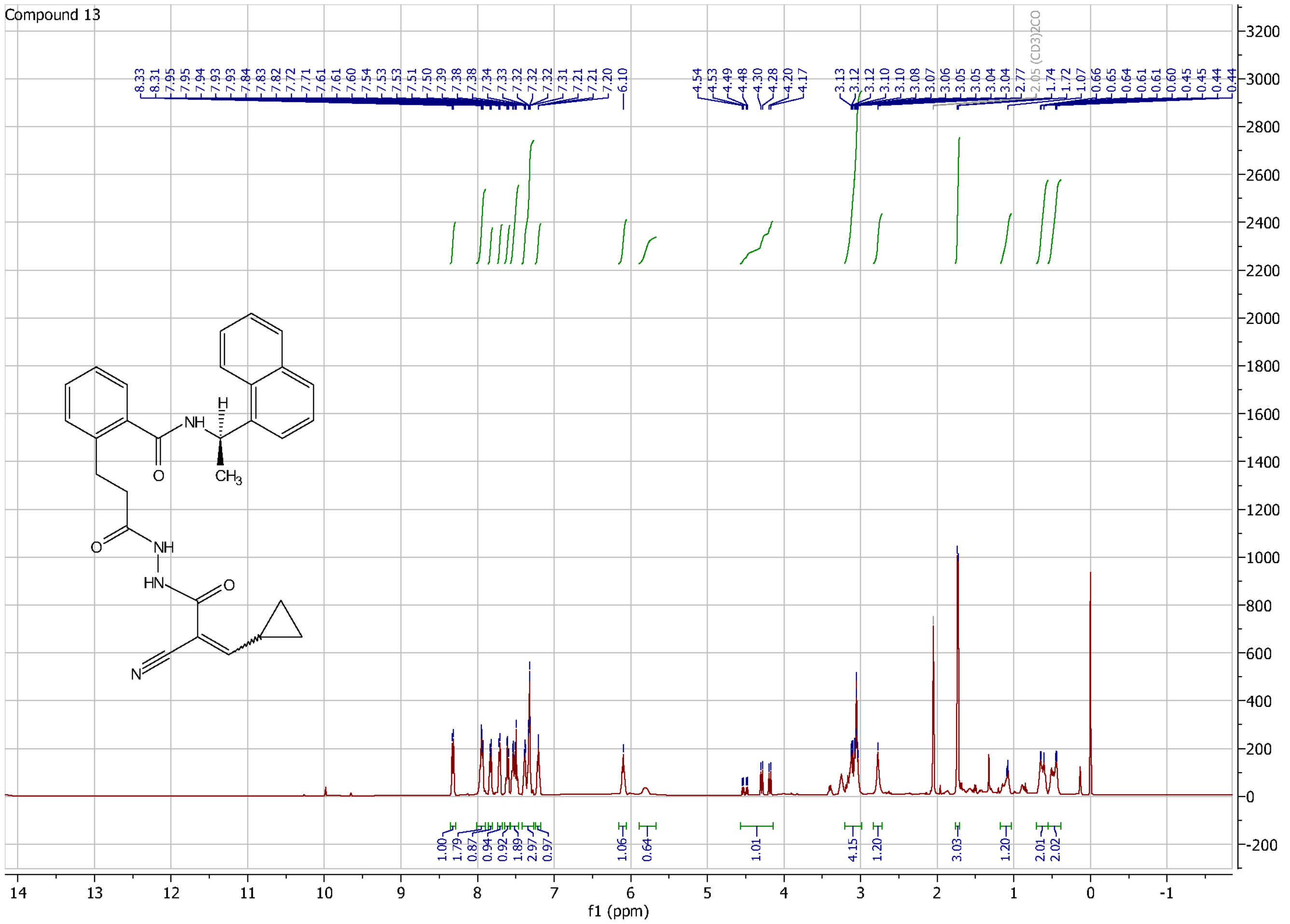

Supplementary Figure 51. ^{1}H NMR spectrum of **13**.

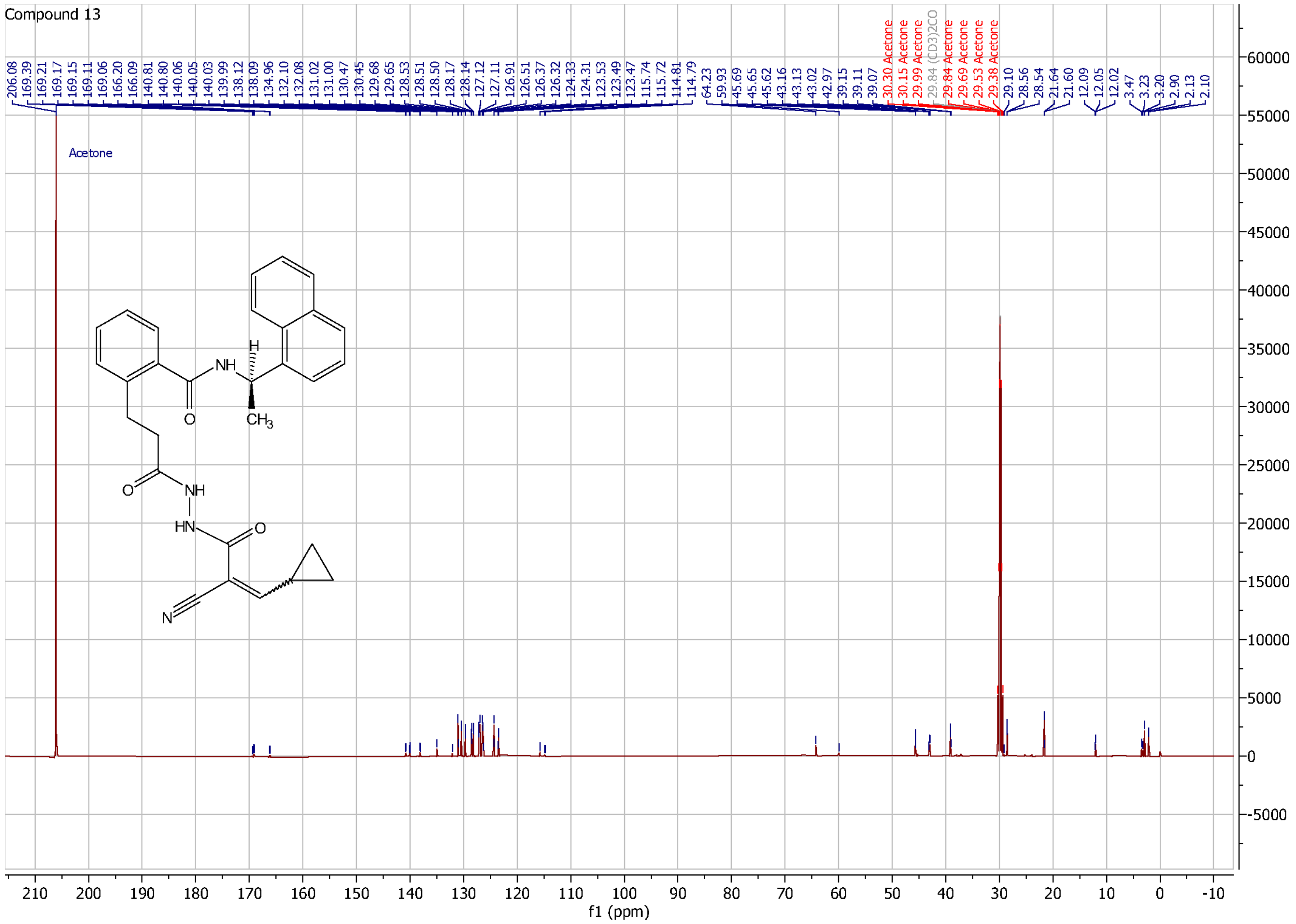

Supplementary Figure 52. ^{13}C NMR spectrum of **13**.
