# Supplementary material for: Potent and selective covalent inhibition of the papain-like protease from SARS-CoV-2
Source: Nat Commun. 2023 Mar 28;14:1733. doi: 10.1038/s41467-023-37254-w (PMC10044120; doi:10.1038/s41467-023-37254-w)
Supplement: Supplementary file 3 — Description of Additional Supplementary Files [file 41467_2023_37254_MOESM3_ESM.pdf]

Description of Additional Supplementary Files

Supplementary Data 1: Noncovalent and covalent docking scores for candidate inhibitors
