# Supplementary material for: Potent and selective covalent inhibition of the papain-like protease from SARS-CoV-2
Source: Nat Commun. 2023 Mar 28;14:1733. doi: 10.1038/s41467-023-37254-w (PMC10044120; doi:10.1038/s41467-023-37254-w)
Supplement: Supplementary file 5 — Reporting Summary [file 41467_2023_37254_MOESM5_ESM.pdf]

nature portfolio

Corresponding author(s): Brian C. Sanders, Soichi Wakatsuki, and Jerry M Parks

Last updated by author(s): 2/24/2023

Reporting Summary

Nature Portfolio wishes to improve the reproducibility of the work that we publish. This form provides structure for consistency and transparency in reporting. For further information on Nature Portfolio policies, see our Editorial Policies and the Editorial Policy Checklist.

Statistics

For all statistical analyses, confirm that the following items are present in the figure legend, table legend, main text, or Methods section.

n/a	Confirmed	
☐	☒	The exact sample size (*n*) for each experimental group/condition, given as a discrete number and unit of measurement
☐	☒	A statement on whether measurements were taken from distinct samples or whether the same sample was measured repeatedly
☒	☐	The statistical test(s) used AND whether they are one- or two-sided<br>*Only common tests should be described solely by name; describe more complex techniques in the Methods section.*
☒	☐	A description of all covariates tested
☒	☐	A description of any assumptions or corrections, such as tests of normality and adjustment for multiple comparisons
☐	☒	A full description of the statistical parameters including central tendency (e.g. means) or other basic estimates (e.g. regression coefficient) AND variation (e.g. standard deviation) or associated estimates of uncertainty (e.g. confidence intervals)
☒	☐	For null hypothesis testing, the test statistic (e.g. F, t, r) with confidence intervals, effect sizes, degrees of freedom and P value noted<br>*Give P values as exact values whenever suitable.*
☒	☐	For Bayesian analysis, information on the choice of priors and Markov chain Monte Carlo settings
☒	☐	For hierarchical and complex designs, identification of the appropriate level for tests and full reporting of outcomes
☒	☐	Estimates of effect sizes (e.g. Cohen's *d*, Pearson's *r*), indicating how they were calculated

Our web collection on statistics for biologists contains articles on many of the points above.

Software and code

Policy information about availability of computer code

Data collection	Bruker Avance III HD 500 MHz NMR spectrometer; Agilent 6470AA Triple Quadrupole LC/MS system; Waters Synapt HDMS QTOF mass spectrometer; Thermo LTQ MS with electrospray ionization; EnVision® 2105 multimode plate reader; Biotek Synergy H1 fluorescence plate reader
Data analysis	Phenix (1.18); AmberTools20; MGLTools (1.5.6); AutoDockFR (1.0); PyMOL (2.5.1 and 2.5.2); OpenBabel (2.4.1); RDKit (2021.09.1 and 2021.09.2), Microsoft Excel (16.62 to 16.68); GraphPad Prism 9; XDS suite (version Feb 5, 2021); MOLREP (11.7.03); Coot (0.9.6); REFMAC 5.8.0267; WinNonlin (version 6.3 to 8.2); MetaSite (6.0.1)

For manuscripts utilizing custom algorithms or software that are central to the research but not yet described in published literature, software must be made available to editors and reviewers. We strongly encourage code deposition in a community repository (e.g. GitHub). See the Nature Portfolio guidelines for submitting code & software for further information.

Data

Policy information about availability of data

All manuscripts must include a data availability statement. This statement should provide the following information, where applicable:

- Accession codes, unique identifiers, or web links for publicly available datasets
- A description of any restrictions on data availability
- For clinical datasets or third party data, please ensure that the statement adheres to our policy

Source data are provided with this paper. Structural data for SARS-CoV-2 PLpro in complex with compound 7 were deposited in the Protein Data Bank (PDB) with

accession code PDB ID 8EUA (https://www.rcsb.org/structure/8EUA). All other data generated in this study are provided in the Supplementary Information and Source Data file. Publicly available datasets used in this study are X-ray crystal structures of SARS-CoV-2 PLpro with accession codes PDB ID: 7JIR, 7CMD, 6WX4, 6W9C, 6WZU, 6XAA, a structure of UCH-L1 with PDB ID: 3KW5, and a structure of USP4 with PDB ID: 2Y6E. Data are available from the corresponding authors upon request.

Human research participants

Policy information about studies involving human research participants and Sex and Gender in Research.

Reporting on sex and gender	N/A
Population characteristics	N/A
Recruitment	N/A
Ethics oversight	N/A

Note that full information on the approval of the study protocol must also be provided in the manuscript.

Field-specific reporting

Please select the one below that is the best fit for your research. If you are not sure, read the appropriate sections before making your selection.

☒ Life sciences ☐ Behavioural & social sciences ☐ Ecological, evolutionary & environmental sciences

For a reference copy of the document with all sections, see nature.com/documents/nr-reporting-summary-flat.pdf

Life sciences study design

All studies must disclose on these points even when the disclosure is negative.

Sample size	No sample size calculations were performed for the in vivo pharmacokinetics study.
Data exclusions	In Figure 5, some measurements were excluded from the plots because they were for low inhibitor concentrations at which the rate of inactivation could not be determined reliably because it was too slow. In Supplementary Figure 6, some data were excluded from plots because they were for high inhibitor concentrations at which the rate could not be determined reliably because it was too fast.
Replication	To ensure reproducibility of the experimental findings, each assay was performed at least two times. We confirm that all attempts at replication were successful.<br>In particular, Michaelis-Menten kinetics assays (Supplementary Figure 4) were performed with n = 2 independent experiments and these data were used to calculate mean values.<br>IC50 measurements with PLpro, peptide substrate, and inhibitor candidates (Table 1, Figure 5, Supplementary Figure 5, Supplementary Figure 6, and Supplementary Figure 7) were performed with n = 2 independent samples for each data point and these data were used to calculate mean values.<br>SARS-CoV-2 PLpro kinact/Ki measurements (Table 1, Figure 5b, and Supplementary Figure 6) were carried out with n = 2 independent samples for each data point (k_obs vs inhibitor concentration) and these data were used to calculate mean values.<br>Deubiquitinase selectivity panel IC50 measurements (Supplementary Table 2) were carried out in quadruplicate and these data were used to calculate mean values.<br>Cell-based (CPE) antiviral activity assays (Table 1, Figure 5, and Supplementary Figure 9) were performed with n = 2 independent samples for each data point. Seven points of inhibitor concentrations were performed. The number of points was chosen to reach 100% cell viability.<br>After HA pulldown, Nsp3 activity assays (Figure 6) were performed in 384-well plates. For compound 7, the complete assay (transfection, pulldown, and assay) was performed in duplicate and these data were used to calculate mean values. For GRL0617, the assay was performed in triplicate and the data were used to calculate mean values.<br>Cell-based (CPE) antiviral assays (Table 2 and Table 3) were performed with n = 2 independent samples and the data were used to calculate mean values.<br>Metabolic stability assays (Supplementary Table 4 and Supplementary Table 5) were performed with n = 2 replicates and the data were used to calculate mean values.<br>In vivo pharmacokinetics (PK) experiments (Supplementary Table 6) involved a total of 48 ICR (CD-1) mice separated into two groups of 24 mice each. One group was used to assess intravenous (IV) PK for compound 7 and the other group was used to assess oral (PO) PK for compound 7. The sample sizes were chosen to allow three biological replicates at eight time points for each group. The data were used to calculate mean values and standard error of the mean (SEM).
Randomization	Mice for in vivo pharmacokinetic experiments were randomly divided into two corresponding groups of 24 mice each for IV and PO assessments. Randomization was not relevant to other experiments because they are not subject to biased interpretation regardless of randomization in sample allocation.
Blinding	For in vivo PK experiments, the mouse groups were randomly divided into two groups. Drug treatment, data collection, and result analysis were blinded, i.e., during the experiments, the person(s) performing the IP or PO injections and weight measurements were not informed which group was treated with drug or water vehicle. This information was revealed after completion of the study. Blinding is not applicable for

in vitro testing of inhibitor potency in cells because the data are not subject to biased interpretation.

Reporting for specific materials, systems and methods

We require information from authors about some types of materials, experimental systems and methods used in many studies. Here, indicate whether each material, system or method listed is relevant to your study. If you are not sure if a list item applies to your research, read the appropriate section before selecting a response.

Materials & experimental systems

n/a	Involved in the study
☐	☒ Antibodies
☐	☒ Eukaryotic cell lines
☒	☐ Palaeontology and archaeology
☐	☒ Animals and other organisms
☒	☐ Clinical data
☒	☐ Dual use research of concern

Methods

n/a	Involved in the study
☒	☐ ChIP-seq
☒	☐ Flow cytometry
☒	☐ MRI-based neuroimaging

Antibodies

Antibodies used	Purified anti-HA.11 Epitope Tag Antibody (Previously Covance catalog # MMS-101P), clone 16B12; The full amino acid sequence is: YPYDVPDYA. Full-length HA-Nsp3 was purified using anti-HA immunoprecipitation (5 mg anti-HA antibody to 1 mg cell lysate), washed 4 times using the lysis buffer and the Nsp3-containing beads (~100 ul bead volume) were resuspended in 1.0 ml enzyme assay buffer (20 mM Tris-HCl, pH 8.0, 0.05% CHAPS, 2 mM beta-mercaptoethanol). 20 ul of the immunoprecipitated Nsp3 beads and the whole cell lysates (30 ug) were run on 8% SDS-PAGE, transferred to a polyvinylidene difluoride (PVDF) membrane, and probed with anti-HA antibody (1:1000 dilution) to detect full-length Nsp3.
Validation	Validation information is available at the following link: https://www.biolegend.com/fr-ch/products/purified-anti-ha-11-epitope-tag-antibody-11374

Eukaryotic cell lines

Policy information about cell lines and Sex and Gender in Research

Cell line source(s)	African green monkey (Vero E6 TMPRSS ACE2) cells (supplier: Dr. Barney Graham, NIH); Vero E6, Human embryonic kidney (HEK293T) from ATCC; human colon, Caco-2 from ATCC
Authentication	All cells from ATCC were provided with authentication, which was performed by STR profiling. Vero E6 TMPRSS ACE2 and HEK293T cells were not authenticated.
Mycoplasma contamination	Cell lines are routinely tested for mycoplasma and are free from mycoplasma.
Commonly misidentified lines (See ICLAC register)	No commonly misidentified cell lines were used.

Animals and other research organisms

Policy information about studies involving animals; ARRIVE guidelines recommended for reporting animal research, and Sex and Gender in Research

Laboratory animals	This study involved 48 male ICR (CD-1) mice (age: 4-6 weeks) that were obtained from BioLasco Taiwan (under license from Charles River Laboratories). All mice weighted 22 +/- 2 g. The age of the mice was not provided. Animals were acclimated for 3 days prior to use and were confirmed with good health. All animals were maintained in a hygienic environment with controlled temperature (20 - 24oC), humidity (30% - 70%) and 12 hours light/dark cycles. Free access to sterilized standard lab diet [MFG (Oriental Yeast Co., Ltd., Japan)] and autoclaved tap water were granted.
Wild animals	This study did not involve wild animals.
Reporting on sex	All mice were male.
Field-collected samples	This study did not involve any field-collected samples.
Ethics oversight	All aspects of the pharmacokinetics work, including housing, experimentation, and disposal of animals were performed in general accordance with the Guide for the Care and Use of Laboratory Animals: Eighth Edition (National Academy Press, Washington, D. C., 2011) in our AAALAC-accredited laboratory animal facility. The animal care and use protocol was reviewed and approved by the IACUC at Pharmacology Discovery Services Taiwan, Ltd.

Note that full information on the approval of the study protocol must also be provided in the manuscript.
